# Protocol to define Sox9 target programs during mouse limb chondrogenesis using scRNA-seq and CUT&RUN integration

**DOI:** 10.1016/j.xpro.2026.104889

**Published:** 2026-10-08

**Authors:** Kohei Kita, Masayasu Sega, Yutaro Uchida, Tomoki Chiba, Takahide Matsushima, Hiroshi Asahara

**Affiliations:** 1 Department of Systems BioMedicine, Graduate School of Medical and Dental Sciences, Institute of Science Tokyo, 1-5-45, Yushima, Bunkyo-ku, Tokyo 113-8510, Japan; 2 Comprehensive Reproductive Medicine, Graduate School of Medical and Dental Sciences, Institute of Science Tokyo, 1-5-45, Yushima, Bunkyo-ku, Tokyo 113-8510, Japan; 3 Department of Molecular and Cellular Biology, The Scripps Research Institute, La Jolla, San Diego, CA 92037, USA

**Keywords:** Cell Biology, Developmental biology, Genomics

## Abstract

Developing tissues comprise heterogeneous cell populations exhibiting stage-dependent transcriptional dynamics. We present a protocol to identify candidate Sox9 target genes in specific murine cell populations by integrating single-cell RNA sequencing (scRNA-seq) with Ty1-tag-based CUT&RUN. We describe steps for embryonic forelimb bud dissection, scRNA-seq and CUT&RUN sample and library preparation, and data processing. We then detail procedures for integrative analysis of stage- and cluster-specific Sox9 targets. The framework may be adaptable to other Sox9-expressing tissues and developmental stages with appropriate optimization.

For complete details on the use and execution of this protocol, please refer to Sega et al.^1^

## Highlights

• Steps for identifying Sox9-high chondroprogenitor populations by scRNA-seq
• Steps for mapping Sox9 binding by CUT&RUN using a Sox9-Ty1 knockin mouse
• Guidance on integrating scRNA-seq and CUT&RUN to identify candidate Sox9 target genes

## Abstract (editor highlights)

Publisher’s note: Undertaking any experimental protocol requires adherence to local institutional guidelines for laboratory safety and ethics.

## Graphical abstract

**Figure:**
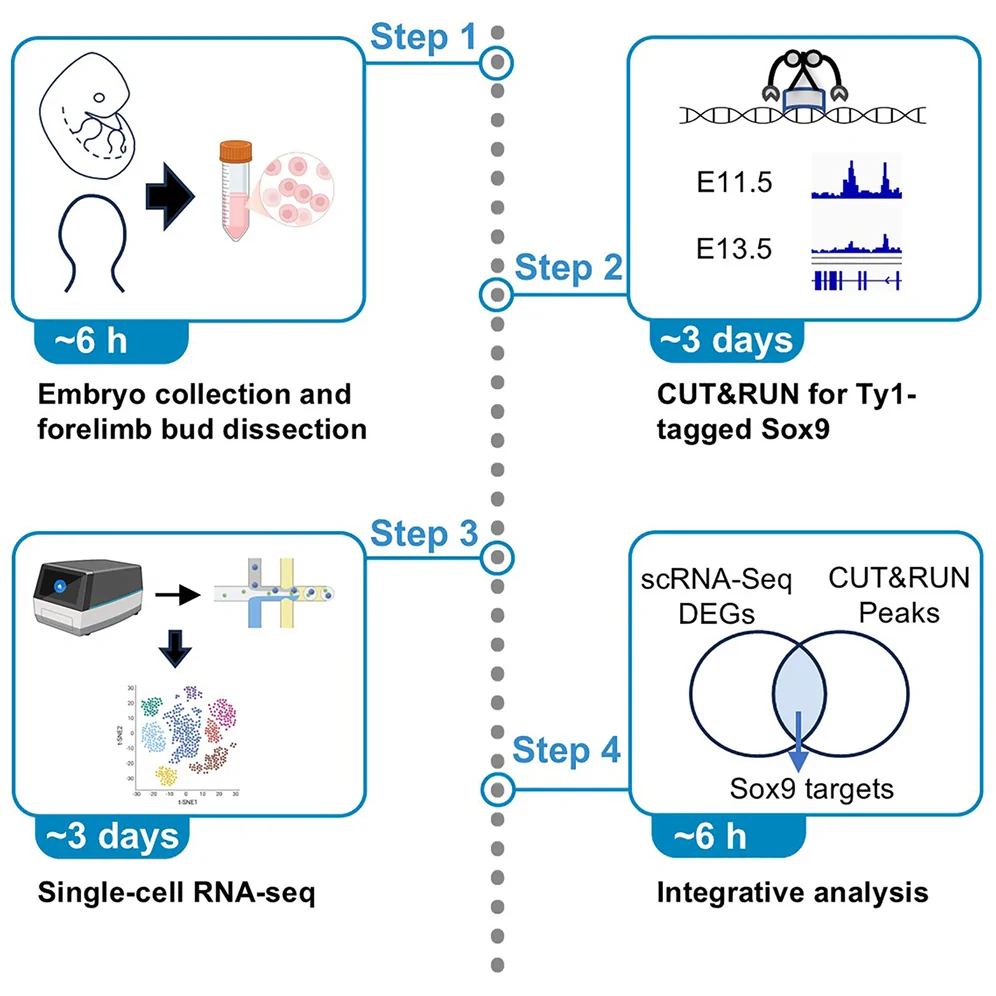

## Before you begin

Mouse limb development is a representative model for understanding morphogenesis, in which diverse cell populations within the limb bud differentiate over time to form distinct patterns along the anteroposterior, proximodistal, and dorsoventral axes. However, it is not straightforward to identify which gene programs a specific transcription factor controls within which cell populations in such heterogeneous tissues. This protocol integrates stage-resolved scRNA-seq and CUT&RUN^2^ to analyze the regulatory networks of Sox9 in the mouse embryonic limb.

In this approach, scRNA-seq is used to characterize cell populations and their transcriptional states, while CUT&RUN is used to map Sox9 genomic binding regions. By combining these two data types, candidate Sox9 target genes can be inferred in the context of specific developmental stages and cell populations. For accurate integrative analysis, synchronized sample collection across developmental stages, consistent forelimb bud dissection boundaries, and optimization of sample preparation conditions for each experimental system are critical.

Before performing CUT&RUN, we recommend consulting the most current CUTANA CUT&RUN protocol and associated library preparation documentation provided by EpiCypher. In particular, conditions for cell or nuclei preparation from embryonic tissue must be optimized in advance according to the target tissue and protein of interest. Before performing scRNA-seq, we recommend consulting the latest official support documentation for the 10x Genomics assay being used.

### Innovation

The innovation of this protocol lies in the integration of stage-resolved scRNA-seq with CUT&RUN using a Sox9-Ty1 knock-in mouse, enabling low-background chromatin profiling and extraction of candidate Sox9 target genes in mouse embryonic forelimbs at both the cell-population and developmental-stage level. While scRNA-seq captures differences in cell states but is limited in direct target identification, CUT&RUN enables high-sensitivity detection of binding regions but provides limited cell-population information. This protocol complementarily combines the strengths of both approaches to allow reproducible analysis of Sox9 cluster-specific transcriptional regulation during development.

Additionally, tag-based CUT&RUN using an anti-Ty1 antibody reduces the background noise and signal variability that can arise when relying on antibodies against endogenous Sox9, enabling stable binding analysis even from limited amounts of embryonic tissue. This design is applicable to other transcription factors for which appropriate tagging and detection conditions can be ensured.

### Institutional permissions (if applicable)

All animal experiments were approved by the Animal Experimental Committee of the Institute of Science Tokyo (A2024-012C5) and conducted in accordance with institutional and national guidelines for the care and use of animals. Researchers implementing this protocol must obtain all necessary approvals in accordance with their institutional and national or regional regulations prior to beginning experiments.

### Maintenance and timed mating of Sox9-Multitag mice

**Timing: 12–14 h for timed mating setup, plus gestation to the desired embryonic stage**

1. Maintain Sox9-Multitag mice under standard housing conditions.

Perform all animal experiments in accordance with protocols approved by the institutional animal care and use committee.

2. To obtain embryos carrying the Sox9-Multitag allele for CUT&RUN, cross Sox9-Multitag homozygous parents with wild-type parents. For scRNA-seq, use embryonic forelimb buds from wild-type ICR mice, which were selected to ensure sufficient embryo and cell yields for single-cell analysis.
3. To obtain embryos at the desired developmental stage, place adult males and females together in the evening.
4. Check for vaginal plugs the following morning. Define the day a vaginal plug is confirmed as embryonic day 0.5 (E0.5).
5. Collect pregnant females once embryos have reached the desired developmental stage for subsequent forelimb bud dissection.

**CRITICAL:** Precise synchronization of embryonic stages across experiments is critical for the reproducibility of this protocol. The timing of mating setup, vaginal plug confirmation, and embryo collection must be standardized across all experiments, and consistent criteria for embryonic stage determination must be applied.

***Note:*** This protocol uses samples from different genetic backgrounds and genotypes for scRNA-seq and CUT&RUN this should be taken into account when interpreting integrative analysis results. The protocol can be applied to other genotypes provided there are no issues with the target allele phenotype or tissue recovery.

***Note:*** The generation of Sox9-Multitag knock-in mice is described in Sega et al.^1^ Researchers wishing to generate this mouse line should refer to the STAR Methods of that publication for oligonucleotide sequences and CRISPR-Cas9 delivery conditions.

### Preparation of reagents and buffers

**Timing: 1–2 h**

Prepare all reagents and buffers required for scRNA-seq, CUT&RUN, and library quality assessment prior to beginning the experiments. Detailed composition, preparation methods, and storage conditions for each buffer are described in the materials and equipment section.

6. Prepare all reagents and buffers before beginning experiments.
  a. Keep all solutions used for embryo collection, forelimb bud dissection, cell dissociation, and CUT&RUN sample preparation on ice until immediately before use.
  b. Prepare buffers containing digitonin and buffers requiring protease inhibitor addition immediately before use.

**CRITICAL:** Standardize reagent storage conditions, buffer temperature, and handling of solutions requiring immediate-use preparation across all experiments. Variability in these conditions may affect cell state and CUT&RUN signal quality.

***Note:*** Handle reagents appropriately under RNase-free or DNase-free conditions as necessary.

### Software setup for computational analysis

**Timing: 0.5–1 day (variable)**

Before beginning scRNA-seq and CUT&RUN data analysis, prepare all necessary software, dependency packages, reference genomes, gene annotation files, and index files.

7. Install Cell Ranger and R/Seurat for scRNA-seq analysis, and FastQC, Bowtie2, SAMtools, and SEACR for CUT&RUN analysis, if not already available. Record the version of each software and confirm proper operation by running test or version-check commands.
8. Obtain reference files required for scRNA-seq and CUT&RUN data analysis, including reference genome sequences, gene annotation files, Cell Ranger references, and Bowtie2 index files. Place the obtained files in a directory accessible from the analysis scripts.
9. Confirm analysis scripts and the computational environment for integrative analysis in advance. Standardize reference genome version, gene annotation version, gene name notation, and sample naming conventions across both scRNA-seq and CUT&RUN analyses.

**CRITICAL:** Differences in software versions or reference data can affect analysis results. In particular, consistent use of reference genome, gene annotation, and gene name notation across scRNA-seq and CUT&RUN integrative analysis is critically important. In this protocol, GRCm39 is used as the reference genome for both scRNA-seq and CUT&RUN analyses to ensure consistency in downstream integration.

***Note:*** The names, versions, and sources of all software used should be listed in the key resources table. Detailed parameters used in each analysis step are provided in step-by-step method details.

## Key resources table

**Table:** 

REAGENT or RESOURCE	SOURCE	IDENTIFIER
**Antibodies**		

Mouse monoclonal anti-Ty1(BB2) (0.5 μg per reaction; 10 μg/mL final)	Thermo Fisher Scientific	Cat#MA5-23513; RRID: AB_2610644;
Rabbit IgG, multiclonal [RM1060] - Isotype control (0.5 μg per reaction; 10 μg/mL final)	Abcam	Cat# ab313801

**Chemicals, peptides, and recombinant proteins**		

Hank’s balanced salt solution (HBSS)	FUJIFILM Wako Pure Chemical Corporation	Cat#082–09865
37% Formaldehyde Solution	FUJIFILM Wako Pure Chemical Corporation	Cat#063–04815
Glycine	FUJIFILM Wako Pure Chemical Corporation	Cat#077–00735
Collagenase D	Roche Diagnostics	Cat#11088866001
Fetal bovine serum (FBS)	Thermo Fisher	Cat#A5256701
Penicillin–streptomycin (PS)	FUJIFILM Wako Pure Chemical Corporation	Cat#168–23191
1 mol/L HEPES-KOH Buffer Solution (pH 7.5)	NACALAI TESQUE	Cat#1563984
1 mol/L HEPES-NaOH, Buffer Solution	NACALAI TESQUE	Cat#1755794
Protease Inhibitor Cocktail Set III DMSO Solution (EDTA free) (×100)	FUJIFILM Wako Pure Chemical Corporation	Cat#169–26063
Digitonin (5%)	Thermo Fisher Scientific	Cat#BN2006
CUTANA pAG-MNase for ChIC/CUT&RUN Workflows	EpiCypher	Cat#15–1016
CUTANA E. coli Spike-in DNA	EpiCypher	Cat#18–1401
Spermidine	Sigma-Aldrich	Cat#85558-1G
Proteinase K	New England Biolabs	Cat#P8107S

**Critical commercial assays**		

Chromium Next GEM Single Cell 3′ Kit v3.1	10x Genomics	Cat#1000269
Chromium Next GEM Chip G Single Cell Kit	10x Genomics	Cat#1000127
3′ Feature Barcode Library Kit	10x Genomics	Cat#1000262
3′ CellPlex Kit Set A	10x Genomics	Cat#1000261
Dual Index Kit TT Set A	10x Genomics	Cat#1000215
Dual Index Kit NN Set A	10x Genomics	Cat#1000243
NEBNext Ultra II DNA Library Prep Kit for Illumina	New England Biolabs	Cat#E7645L

**Deposited data**		

Analyzed data (scRNA-seq; example dataset)	This paper	BioProject: PRJDB37869; GEA: E-GEAD-1154
Analyzed data (CUT&RUN example dataset)	This paper	BioProject: PRJDB37870; GEA: E-GEAD-1157

**Experimental models: organisms/strains**		

Mouse: C57BL/6J Jms Slc, wild-type embryos, E11.5, sex not determined	Sankyo Lab Service	–
Mouse: Sox9-Multitag heterozygous knock-in embryos, C57BL/6J background, E11.5 and E13.5, sex not determined	Sega et al.^1^	–
Mouse: Slc:ICR, wild-type embryos, E9.5–E13.5, sex not determined	Sankyo Lab Service	–

**Oligonucleotides**		

Genotyping forward primer: 5′-TCCCTTCCATCCCGCAGA-3′	Sega et al.^1^	–
Genotyping reverse primer: 5′-AGATCAGCTCTGTCACCATAGC-3′	Sega et al.^1^	–

**Software and algorithms**		

Cell Ranger v8.0.1	10x Genomics	https://www.10xgenomics.com/support/software/cell-ranger/downloads
Mouse reference genome GRCm39	10x Genomics	https://www.10xgenomics.com/support/software/cell-ranger/downloads
Seurat v5.1.0	Hao et al.^3^	https://satijalab.org/seurat/
R v4.4.0	R Foundation for Statistical Computing	https://www.r-project.org/
TrimGalore v0.6.10	The Babraham Institute	https://github.com/FelixKrueger/TrimGalore
FastQC v0.12.1	Andrews^4^	https://github.com/s-andrews/FastQC
GENCODE mouse annotation, release vM33 (GRCm39)	GENCODE	https://www.gencodegenes.org/mouse/release_M33.html
txdbmaker v1.6.2	Bioconductor	https://bioconductor.org/packages/txdbmaker
*Escherichia coli* K-12 substr. MG1655 reference genome (ASM584v2)	NCBI RefSeq	RefSeq: GCF_000005845.2; https://www.ncbi.nlm.nih.gov/datasets/genome/GCF_000005845.2/
Bowtie2 v2.5.4	Langmead et al.^5^	https://bowtie-bio.sourceforge.net/bowtie2/
SAMtools v1.22.1	Danecek et al.^6^	https://www.htslib.org/
Picard v3.4.0	Broad Institute	https://github.com/broadinstitute/picard
deepTools v3.5.5	Ramírez et al.^7^	https://github.com/deeptools/deepTools
bedtools v2.31.1	Quinlan et al.^8^	https://github.com/arq5x/bedtools2
SEACR v1.3	Meers et al.^9^	https://github.com/FredHutch/SEACR
HOMER v5.1	Heinz et al.^10^	http://homer.ucsd.edu/homer/
ChIPseeker v1.46.1	Yu et al.^11^	https://github.com/YuLab-SMU/ChIPseeker
org.Mm.eg.db v3.22.0	Bioconductor	https://bioconductor.org/packages/release/data/annotation/html/org.Mm.eg.db.html

**Other**		

Stereo Microscope	Nikon Corporation	Cat#SMZ745T
1.5-mL tubes	BIO-BIK Ina Optica	Cat#LT-0150
Student Iris Scissors, 11.5 cm, straight, sharp/sharp	Fine Science Tools	Cat#91460–11
Proteosave SS 1.5 mL low-protein-binding tubes	Sumitomo Bakelite	Cat#MS-4265M
Eppendorf ThermoMixer C	Eppendorf	Cat#5382000023
EASYstrainer for 50 mL Tubes, 70 μm	Greiner Bio-One	Cat#542070
Countess II FL automated cell counter	Thermo Fisher Scientific	Cat# AMQAF1000
Countess Cell Counting Chamber Slides	Thermo Fisher Scientific	Cat# C10228
Chromium iX	10x Genomics	Cat#1000328
BioMag Plus Concanavalin A (ConA) beads	Bangs Laboratories	Cat#BP531
SPRIselect	Beckman Coulter	Cat#B23318
Qubit Flex Fluorometer	Thermo Fisher Scientific	Cat#QFlex01-S3
Invitrogen Qubit dsDNA Quantification Assay Kits	Thermo Fisher Scientific	Cat#Q32854
4150 TapeStation	Agilent	Cat#G2992AA
High Sensitivity D1000 ScreenTape	Agilent	Cat#5067–5584
High Sensitivity D1000 Reagents	Agilent	Cat#5067–5585

## Materials and equipment

Prepare the following buffers fresh or according to the storage conditions specified. All buffers should be prepared using ultrapure water unless otherwise specified.

**Table:** Bead Activation Buffer

Reagent	Final concentration	Amount
1 M HEPES-KOH pH 7.5	20 mM	200 μL
1 M KCl	10 mM	100 μL
0.1 M CaCl_2_	1 mM	100 μL
1 M MnCl_2_	1 mM	10 μL
ddH_2_O	–	Add to a final volume of 10 mL

Store at 4°C for up to 6 months.

**Table:** NE Buffer Stock

Reagent	Final concentration	Amount
1 M HEPES-KOH pH 7.5	20 mM	1 mL
1 M KCl	10 mM	500 μL
100% Triton X-100	0.1%	50 μL
100% Glycerol	20%	10 mL
ddH_2_O	–	Add to a final volume of 50 mL

Store at 4°C for up to 6 months.

**Table:** NE Buffer

Reagent	Final concentration	Amount
NE Buffer Stock	–	1,473.22 μL
100× Protease Inhibitor Cocktail	1×	15 μL
63.7 mM Spermidine	0.50 mM	11.78 μL
Total	–	1,500 μL

Prepare fresh.

***Note:*** Prepare 63.7 mM Spermidine working solution from Spermidine powder dissolved in ddH_2_O. Prepare NE Buffer immediately before use.

**Table:** Pre-XL Wash Buffer Stock

Reagent	Final concentration	Amount
1 M HEPES-NaOH pH 7.5	20 mM	1 mL
5 M NaCl	150 mM	1.5 mL
100% Triton X-100	1%	500 μL
10% SDS	0.05%	250 μL
ddH_2_O	–	up to 50 mL

Store at 4°C for up to 6 months.

**Table:** XL Wash Buffer

Reagent	Final concentration	Amount
Pre-XL Wash Buffer Stock	–	9,821.51 μL
100× Protease Inhibitor Cocktail	1×	100 μL
63.7 mM Spermidine	0.50 mM	78.49 μL
Total	–	10 mL

Prepare fresh.

***Note:*** XL Digitonin Buffer: Add 20 μL of 5% Digitonin per 10 mL XL Wash Buffer. Prepare immediately before use. XL Antibody Buffer: Add 2 μL of 0.5 M EDTA (pH 8.0) per 500 μL XL Digitonin Buffer. Prepare immediately before use.

**Table:** Stop Buffer

Reagent	Final concentration	Amount
5 M NaCl	340 mM	20.4 μL
0.5 M EDTA	20 mM	12 μL
0.5 M EGTA	4 mM	2.4 μL
20 mg/mL RNase A	50 μg/mL	0.75 μL
15 mg/mL Glycogen	0.05 mg/mL	1 μL
ddH_2_O	–	263.45 μL
Total	–	300 μL

Prepare fresh.

***Note:*** Prepare immediately before use. Add E. coli spike-in DNA at 0.5 ng per 5×10^5^ input cells immediately before use.

## Step-by-step method details

### Embryo collection and forelimb bud dissection for CUT&RUN

**Timing: 3–4 h (depending on the number of embryos recovered)**

This step describes the collection of mouse embryos and dissection of forelimb buds for downstream CUT&RUN analysis.

1. To ensure sufficient cell numbers for CUT&RUN, perform timed matings using at least 2 females per experiment and pool embryos from the two resulting litters.
2. Prepare 1.5 mL tubes for limb bud collection and fill each tube with 500 μL of HBSS.
3. Euthanize the pregnant mouse by cervical dislocation.
  a. Excise the uterus as a single unit.
  b. Transfer it to a 100 mm dish containing ice-cold Hank’s balanced salt solution (HBSS) supplemented with 1% penicillin-streptomycin (PS).
4. Using ophthalmic scissors, incise the uterine wall and recover the embryos. Transfer the recovered embryos to a separate dish and dissect under a stereomicroscope (Figure 1A).
  a. Remove the placenta.
  b. If needed, collect the amniotic membrane in a separate tube and store at 4°C for up to 6 h before genotyping.

**Figure 1:**
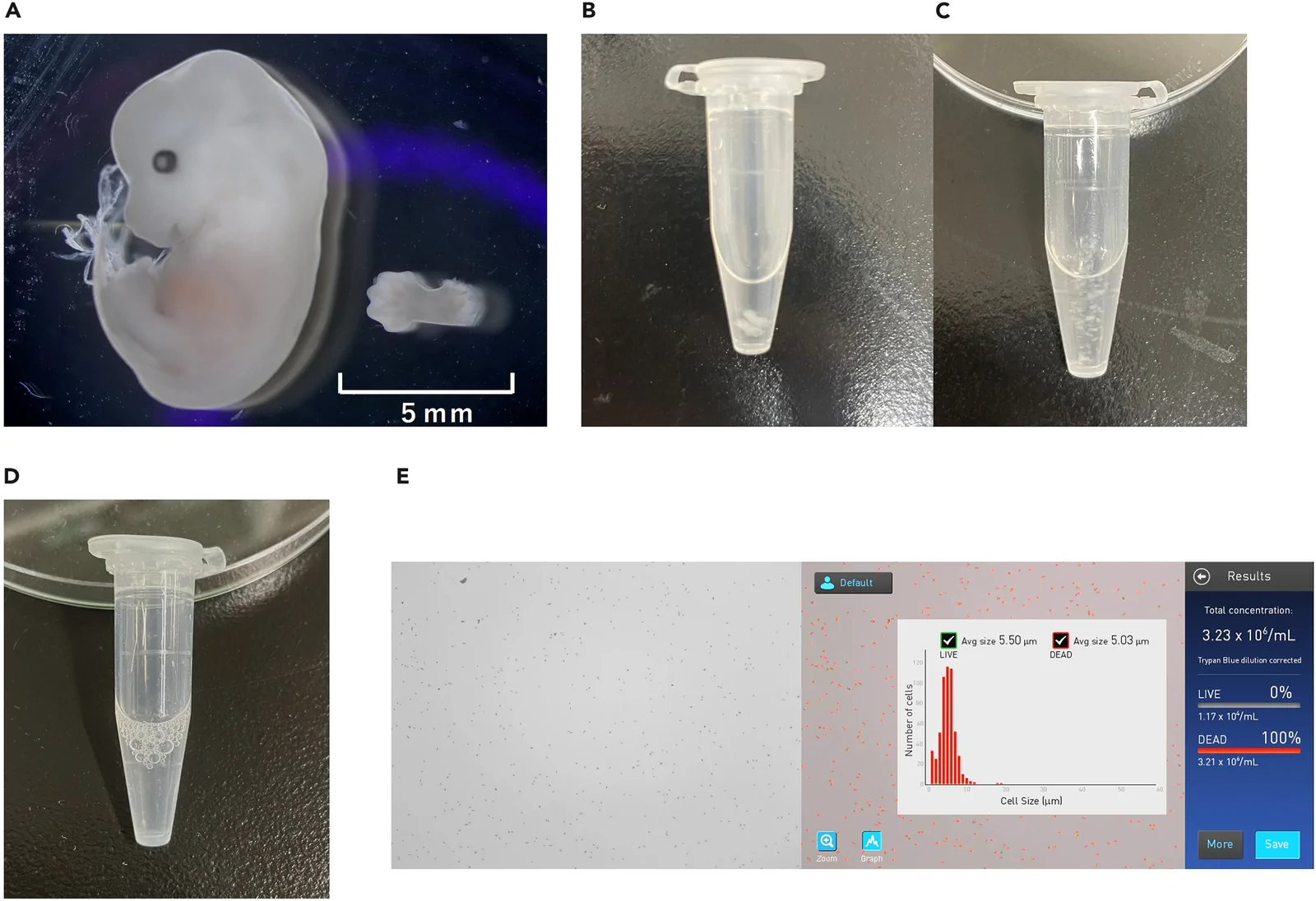
Isolation and dissociation of mouse embryonic forelimb buds for CUT&RUN and scRNA-seq (A) Representative image of an E13.5 mouse embryo with an isolated forelimb bud (right). Embryo collection was performed using the step-by-step protocol of Yea et al.,^12^ originally developed for hindlimb collection. Scale bar, 5 mm. (B) Dissected forelimb buds are collected into a 1.5 mL tube containing HBSS. (C) Forelimb buds are minced into approximately 10 pieces for E11.5 samples and approximately 20 pieces for E13.5 samples using micro-scissors. Avoid over-mincing, as excessive fragmentation can lead to cell aggregation and reduce dissociation efficiency. (D) After collagenase D treatment, the suspension appears as a turbid white liquid, indicating successful tissue dissociation. (E) Representative automated cell counting result after formaldehyde fixation and collagenase D dissociation of two E13.5 forelimbs (Countess II FL, brightfield mode with trypan blue). Left, brightfield counting image; right, cell size distribution. Total concentration was 3.23 × 10^6^ cells/mL with a mean diameter of 5.0 μm. Note that fixed cells appear as 100% dead because trypan blue enters through permeabilized membranes; viability cannot be assessed on fixed samples. The narrow size distribution centered at approximately 5 μm, with few events above 10 μm, indicates that the suspension consists predominantly of single cells with minimal aggregation.
5. Carefully dissect the forelimb buds from each embryo and collect them into the prepared 1.5 mL tubes labeled for CUT&RUN (Figure 1B).
6. Immediately proceed to the subsequent fixation and cell preparation step with the collected forelimb buds.

***Note:*** Approximately 1.0–1.5 × 10^5^ cells are typically recovered from the two forelimb buds of one E11.5 embryo; therefore, at least 4–5 embryos are required to obtain 5 × 10^5^ cells for one CUT&RUN reaction. Approximately 3 × 10^6^ cells are typically recovered from the two forelimb buds of one E13.5 embryo. In our experiments, embryos from multiple litters were pooled as needed depending on the number of CUT&RUN reactions.

**CRITICAL:** Perform all operations from embryo collection through forelimb bud dissection rapidly under cold conditions to minimize changes in sample state.

**CRITICAL:** Variability in developmental stage or in the extent of forelimb bud excision can affect cell composition and chromatin state. Select embryos using the same criteria at each developmental stage, and standardize the forelimb bud excision boundaries, sample handling conditions, and time to downstream processing across experiments. Exclude embryos that show clearly delayed development.

### Fixation and generation of dissociated cells from embryonic forelimb buds for CUT&RUN

**Timing: 2–3 h**

This step fixes embryonic forelimb bud tissue and generates dissociated cells for subsequent nuclei extraction and CUT&RUN analysis. This workflow is a tissue-adapted, fixation-assisted modification optimized for embryonic forelimb buds.

7. On ice, mince the forelimb bud tissue using micro-scissors (Figure 1C).
  a. Cut each limb bud into small pieces, approximately 10 pieces for E11.5 samples and approximately 20 pieces for E13.5 samples, depending on tissue size.

**CRITICAL:** Do not over-mince the tissue. Excessive mincing can cause fixed tissue fragments to adhere to tube walls, reducing recovery efficiency in subsequent steps.

8. Centrifuge samples at 1,000 × *g* for 5 min at 4°C and remove the supernatant.

***Note:*** If fixed cells or tissue fragments remain attached to the tube wall and a clear pellet is not obtained at 1,000 × *g*, the centrifugation force can be increased up to 2,300 × *g*. In our experiments, centrifugation at 2,300 × *g* improved sample recovery without apparent adverse effects on downstream CUT&RUN performance.

9. Add 500 μL of 1% formaldehyde in HBSS and fix at 20°C–25°C temperature for 10 min.
10. Tap the tube gently, then pulse-centrifuge at 2,000 × *g* for 2 s at 20°C–25°C to collect droplets at the bottom of the tube.
11. Add 26.3 μL of 2.5 M glycine to quench the fixation reaction.
12. Centrifuge samples at 1,000 × *g* for 5 min at 4°C and remove the supernatant.
13. Resuspend the pellet in 1 mL of HBSS, centrifuge at 1,000 × *g* for 5 min at 4°C, and remove the supernatant.
14. Resuspend the pellet in 500 μL of HBSS, and add 12.8 μL of collagenase D stock solution (100 mg/mL) to achieve a final concentration of 2.5 mg/mL.
15. Incubate samples at 37°C with shaking at 1,000 rpm on an Eppendorf ThermoMixer C for 1–2 h (Figure 1D).
  a. Tap the tube gently once every 30 min and continue shaking until cells are sufficiently dissociated.
16. Quench the dissociation reaction and prepare the cell suspensions for immediate filtration.
  a. Pulse-centrifuge the samples at 2,000 × *g* for 2 s at 20°C–25°C to collect droplets at the bottom of the tubes.
  b. Place a 70 μm cell strainer on a 50 mL tube.
  c. Add 500 μL of HBSS supplemented with 10% FBS and 1% PS to quench the dissociation reaction.
  d. Gently pipette the resulting 1 mL cell suspension to homogenize.
17. Using the same pipette, immediately pass the entire 1 mL cell suspension from each tube belonging to the same developmental stage and genotype through the cell strainer.

***Note:*** Use a separate cell strainer and 50 mL tube for each developmental stage and genotype to prevent cross-contamination and unintended pooling between groups.

18. Wash the cells remaining on the original tube wall with 1,000 μL of HBSS supplemented with 10% FBS and 1% PS, and collect into the same 50 mL tube.
19. Wash the cell strainer with 1 mL of HBSS supplemented with 10% FBS and 1% PS, and add the wash solution to the same 50 mL tube.
20. Centrifuge samples at 2,300 × *g* for 10 min at 4°C and remove the supernatant.
21. Resuspend the pellet in 1,000 μL of HBSS supplemented with 1% PS in the same 50 mL tube.
22. Centrifuge samples at 2,300 × *g* for 5 min at 4°C and remove the supernatant.
23. Finally, resuspend the pellet in 1,000 μL of HBSS supplemented with 1% PS.
24. Take 10 μL of the cell suspension and measure the cell count using a cell counter.

***Note:*** Approximately 1.0–1.5 × 10^5^ cells are typically recovered from the two forelimb buds of one E11.5 embryo, whereas approximately 3 × 10^6^ cells are typically recovered from the two forelimb buds of one E13.5 embryo (Figure 1E). For E11.5 samples, at least 4–5 embryos are therefore required to obtain 5 × 10^5^ cells for one CUT&RUN reaction. In this protocol, 5 × 10^5^ cells are used per CUT&RUN reaction (see Step 32).

25. Proceed with the cell suspension to ConA bead activation and subsequent nuclear extraction and ConA bead binding (Steps 26–37).

**CRITICAL:** Maintain consistent fixation time, collagenase D concentration, and dissociation time across all samples. Excessive fixation or insufficient dissociation may affect downstream CUT&RUN signal and reproducibility.

**CRITICAL:** When the negative control is prepared from a separate pool of embryos, process it in parallel with the target samples using identical fixation time, collagenase D concentration, dissociation time, and cell input.

***Note:*** This procedure was optimized using embryos ranging from E11.5 to E13.5. Adjustment of tissue volume and dissociation time may be required for application to different developmental stages.

***Note:*** Each pooled cell suspension prepared as described above — comprising forelimb buds from multiple embryos of the same developmental stage and genotype, obtained from two or more litters — constitutes one biological replicate. Pooling is required to reach the cell input needed for a CUT&RUN reaction at earlier stages (see Step 24), but it averages inter-individual variation within a replicate and does not substitute for independent biological replicates.

***Note:*** In this protocol, the IgG control was prepared from a separate pool of wild-type embryos rather than from an aliquot of the tagged sample. In the example dataset, one Sox9 (Ty1) library was prepared per stage (E11.5 and E13.5), together with a single IgG control library from E11.5 wild-type tissue; peak overlap between independent replicates was therefore not assessed (see limitations).

### Con A bead activation

**Timing: ∼30 min**

This step activates ConA beads for subsequent binding of nuclei prepared from dissociated forelimb bud cells, following the approach described by Janssens et al.^13^ Uniform bead activation is important for reproducible nuclei binding.

26. Gently invert the BioMag Plus Concanavalin A beads to mix, and aliquot 11 μL per reaction into 1.5 mL tubes.
27. Place the tubes on a 1.5 mL magnetic stand, allow the slurry to clear, and remove the supernatant.
28. Remove the tubes from the magnet, add 100 μL of cold Bead Activation Buffer per sample, and mix by pipetting.
29. Place the tubes back on the magnet and remove the supernatant once the slurry has cleared.
  a. Perform a total of two washes with Bead Activation Buffer (repeat Steps 28–29 once).
30. After washing, resuspend the beads in 11 μL of cold Bead Activation Buffer per sample.
31. Aliquot 10 μL of the activated bead slurry per reaction into 8-strip tubes and keep on ice.

**CRITICAL:** Wash and activate the ConA beads thoroughly, and take care to prevent the bead slurry from drying. Bead loss or drying can reduce downstream nuclei binding efficiency.

**Pause point:** Activated ConA beads can be kept on ice (0°C–4°C) for up to 30 min while cell numbers are confirmed and the cells are processed through Steps 32–35. Do not allow the beads to dry during this period.

### Nuclear extraction and binding to activated Con A beads

**Timing: ∼30 min**

This step prepares nuclei from dissociated embryonic forelimb bud cells and binds them to activated ConA beads for downstream antibody incubation. Consistent cell input and careful handling of nuclei and beads improve reproducibility.

32. From the cell suspension prepared in Step 25, collect 500,000 cells per sample into 1.5 mL tubes.

***Note:*** The use of flat-bottom 1.5 mL low-protein-binding tubes (e.g., Proteosave SS, Sumitomo Bakelite, Cat#MS-4265M) is recommended to improve visibility and recovery of the nuclear pellet.

33. Centrifuge cells at 600 × *g* for 3 min at 4°C and remove the supernatant.
  a. Confirm that a pellet has formed at the bottom of the tube after centrifugation (Figure 2).

**Figure 2:**
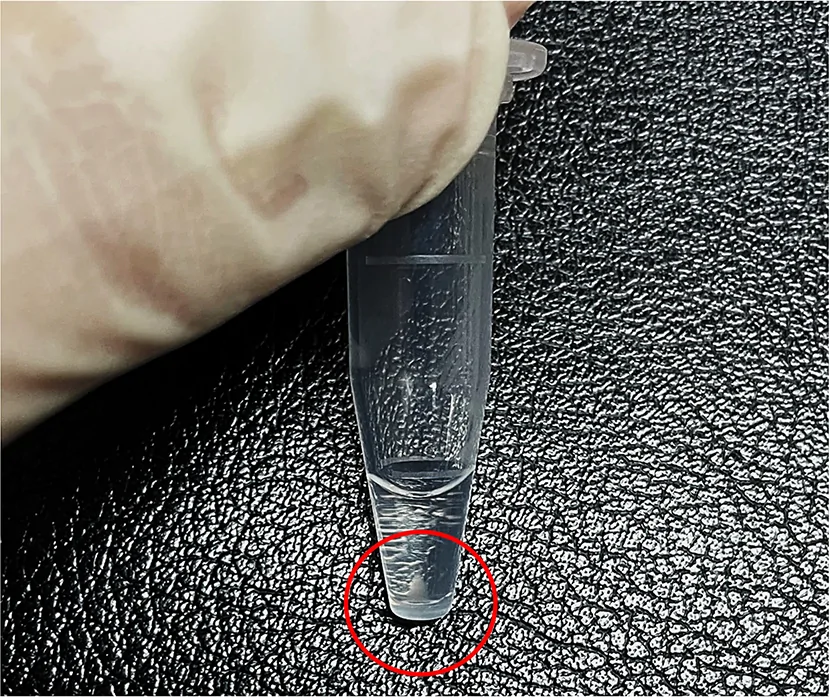
Cell pellet after centrifugation of fixed forelimb bud cells A small but visible white pellet (red circle) is observed at the bottom of the 1.5 mL tube after centrifugation of approximately 5 × 10^5^ fixed cells. Successful pellet formation confirms adequate cell recovery before proceeding to Concanavalin A bead binding.
34. Resuspend the cell pellet in 100 μL of cold NE Buffer per sample for nuclear extraction, then centrifuge at 600 × *g* for 3 min at 4°C and remove the supernatant.
35. After washing, resuspend the nuclei in 100 μL of cold NE Buffer per sample and gently pipette to homogenize.
36. Add 100 μL of the nuclear suspension to each 8-strip tube containing 10 μL of activated ConA beads.
37. Gently vortex (Vortex-Genie, setting 7) and incubate at 20°C–25°C for 10 min to bind the nuclei to the ConA beads.

**CRITICAL:** Match input cell numbers as closely as possible across all samples. Nuclei after NE Buffer treatment are fragile; avoid excessive pipetting or vigorous vortexing. Since bead drying during the binding step can reduce recovery and reproducibility, proceed to the next step quickly after removing the supernatant.

***Note:*** In this protocol, nuclei are prepared from the dissociated cell suspension obtained in Step 23–25 using NE Buffer and then bound to ConA beads. All subsequent CUT&RUN reactions are performed using bead-bound nuclei.

### Primary antibody binding to bead-bound nuclei

**Timing: ∼30 min setup + 12–16 h incubation**

This step binds the primary antibody to bead-bound nuclei before pAG-MNase incubation. Uniform antibody binding is essential for specific and reproducible CUT&RUN signals.

38. Place the 8-strip tubes containing bead-bound nuclei on a magnetic stand, allow the slurry to clear, and remove the supernatant.
39. Add 50 μL of cold XL Antibody Buffer to each sample and gently vortex to resuspend the beads uniformly.
40. Add 0.5 μg of primary antibody to each sample and gently vortex to mix.
  a. For the target reaction, add 0.5 μg of the anti-Ty1 antibody.
  b. For the negative-control reaction, add 0.5 μg of the IgG control antibody instead of the anti-Ty1 antibody.
41. Incubate the 8-strip tubes on a nutator at 4°C for 12–16 h to allow the primary antibody to react with the bead-bound nuclei.

**CRITICAL:** During the antibody reaction, gently rock the tubes on a nutator. Do not perform end-over-end rotation or inversion mixing. Drying of the ConA beads or their attachment to tube walls can reduce recovery efficiency.

**Pause point:** Incubate the samples on a nutator at 4°C for 12–16 h. After completing the incubation, proceed directly to Step 42.

### Binding pAG-MNase to antibody-bound nuclei

**Timing: ∼30 min**

This step binds pAG-MNase to antibody-bound nuclei immobilized on ConA beads. Uniform pAG-MNase binding is important for efficient and reproducible chromatin cleavage.

42. After the primary antibody reaction, pulse-centrifuge the 8-strip tubes at 2,000 × *g* for 2 s at 20°C–25°C, place them on a magnetic stand, allow the slurry to clear, and remove the supernatant.
43. With the tubes on the magnet, add 200 μL of cold XL Digitonin Buffer to each sample and remove the supernatant.
  a. Perform a total of two washes with XL Digitonin Buffer (repeat this step once).
44. After washing, add 50 μL of cold XL Digitonin Buffer to each reaction and gently vortex (setting 7) to resuspend the beads.
45. Add 2.5 μL of pAG-MNase (20× stock) to each CUT&RUN reaction and gently vortex (setting 7) to mix.
46. Incubate samples at 20°C–25°C for 10 min to allow pAG-MNase to react with the antibody-bound complex.
47. Pulse-centrifuge the 8-strip tubes at 2,000 × *g* for 2 s at 20°C–25°C, then place them back on the magnetic stand. Once the slurry has cleared, remove the supernatant.
48. With the tubes on the magnet, add 200 μL of cold XL Digitonin Buffer directly to the beads in each reaction and remove the supernatant.
  a. Perform a total of two washes (repeat this step once).
  b. After the second wash, remove the supernatant completely.
49. Remove the tubes from the magnet, add 50 μL of cold XL Digitonin Buffer to each reaction, and gently vortex (setting 7) to resuspend.

**CRITICAL:** The reaction time and temperature after pAG-MNase addition affect downstream cleavage efficiency and background. Maintain reaction conditions strictly consistent across all samples.

### Targeted chromatin digestion and release of CUT&RUN DNA

**Timing: ∼3 h + 12–16 h Proteinase K digestion**

This step initiates targeted chromatin cleavage and releases fragmented CUT&RUN DNA into the supernatant. Proper control of CaCl_2_ activation, digestion time, and stop conditions is critical for obtaining high-quality signal.

50. Place the 8-strip tubes on ice, add 1 μL of 100 mM CaCl_2_ to each reaction, and gently vortex (setting 7) to mix.
51. Place the tubes on a nutator at 4°C with the lids loosely closed (not fully sealed) and incubate for 2 h to perform targeted chromatin digestion.
52. Prepare the Stop Buffer Master Mix immediately before use, adding 0.5 ng of E. coli spike-in DNA per 500,000 input cells.
53. Add 33 μL of Stop Buffer Master Mix to each reaction and gently vortex (setting 7) to mix.
54. Incubate the samples at 37°C for 10 min in a thermal cycler to promote release of the cleaved fragments.
55. Pulse-centrifuge the samples at 2,000 × *g* for 2 s at 20°C–25°C to collect the beads and buffer at the bottom of the tubes.
56. Place the 8-strip tubes on a magnetic stand and allow the slurry to clear.
57. Transfer 84 μL of the supernatant containing the fragmented CUT&RUN DNA to a new tube.
58. To each supernatant, add 0.8 μL of 10% SDS and 1 μL of 20 μg/μL Proteinase K, and mix by pipetting.
59. Incubate the samples at 55°C for 12–16 h to allow protein digestion.

**CRITICAL:** The reaction time and temperature after CaCl_2_ addition directly affect cleavage efficiency and background. Maintain reaction conditions strictly consistent across all samples.

***Note:*** Use a Stop Buffer Master Mix in which the E. coli spike-in DNA has been pre-added. This helps maintain a consistent spike-in signal for downstream normalization.

**Pause point:** Proteinase K digestion can be continued at 55°C for 12–16 h. After completing the incubation, proceed directly to Step 60.

### DNA recovery and library preparation for CUT&RUN samples

**Timing: ∼5 h**

This step purifies CUT&RUN DNA fragments released into the supernatant and converts them into sequencing libraries. Careful bead cleanup and library preparation are important for maximizing DNA recovery and minimizing technical variability.

60. To each Proteinase K-treated sample from Step 59, add 153 μL of SPRIselect beads, corresponding to approximately 1.8× the sample volume.
61. Mix thoroughly by pipetting.
62. Incubate at 20°C–25°C for 5 min to allow DNA to bind to the beads.
63. Place the tubes on a magnetic stand, wait 5 min for the slurry to clear, and carefully remove the supernatant.
64. Add 200 μL of 80% ethanol to each sample, incubate for 30s, and remove the supernatant.
  a. Perform a total of two ethanol washes.
65. Taking care not to over-dry the beads, add 52 μL of 10 mM Tris-HCl (pH 8.0) to each sample to elute the DNA. Mix by pipetting and incubate for 2 min.
66. Place the tubes on a magnetic stand and transfer 51 μL of the supernatant to a new PCR tube.
67. Use 1 μL of the purified CUT&RUN DNA to quantify the DNA concentration with a Qubit fluorometer.
68. Prepare CUT&RUN DNA libraries using the NEBNext Ultra II DNA Library Prep Kit for Illumina according to the manufacturer’s instructions (https://www.neb.com/en/products/e7645-nebnext-ultra-ii-dna-library-prep-kit-for-illumina). The following parameters are specific to CUT&RUN libraries:
  a. Input DNA volume: Use the full eluate (50 μL) from Step 66.
    ***Note:*** The total DNA input is typically < 50 ng.
  b. Adapter dilution: For input <5 ng, dilute the NEBNext Adaptor 25-fold (final 0.6 μM). For input 5–100 ng, dilute 10-fold (final 1.5 μM).
  c. Post-ligation cleanup: Perform cleanup (Section 3B in the NEB manual; 0.9× SPRIselect beads), not size selection (Section 3A).
  d. PCR cycle number: Use 9–13 cycles for typical CUT&RUN library inputs (adjust according to NEB Table 4.1 based on Qubit quantification).
  e. Final cleanup: Perform 0.9× SPRIselect cleanup as described in Section 5 of the NEB manual; elute in 33 μL of 10 mM Tris-HCl (pH 8.0).
    **CRITICAL:** PCR cycle number significantly affects library quality. Over-amplification can introduce duplicates and bias. Adjust the cycle number based on the input DNA amount measured by Qubit.
    ***Note:*** For samples with very low DNA recovery (e.g., negative controls), additional cycles may be needed to obtain sufficient library yield for sequencing.
69. Assess the quality and size distribution of the purified library using 1 μL on the Agilent 4150 TapeStation with High Sensitivity D1000 ScreenTape and Reagents.
  a. Confirm that the library shows a major peak between approximately 250 and 700 bp with minimal adapter-dimer contamination at approximately 150 bp (Figure 3A).

**Figure 3:**
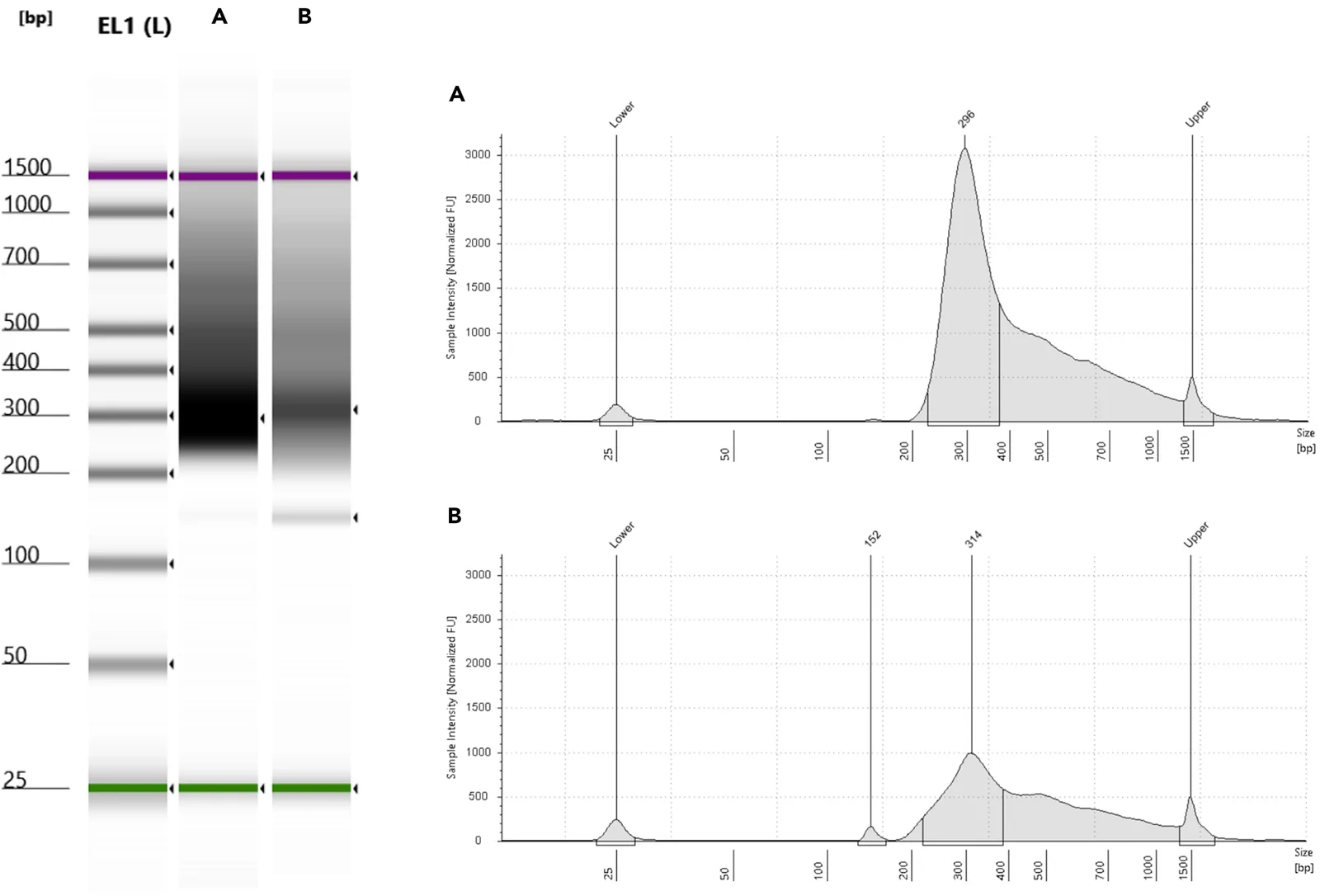
Quality assessment of CUT&RUN libraries by TapeStation Quality assessment using the Agilent 4150 TapeStation with High Sensitivity D1000 ScreenTape. Left, gel image; right, electropherograms. (A) A successful CUT&RUN library shows a major peak at approximately 296 bp without significant adapter dimer (∼150 bp) contamination, indicating a high-quality library suitable for direct sequencing. (B) A suboptimal library shows a prominent adapter dimer peak at approximately 152 bp and a weaker main peak at 314 bp. Lower (25 bp) and Upper (1500 bp) markers are shown as references.
  b. Calculate the molar concentration of library fragments within the 200–700 bp region for downstream pooling.
  c. If a prominent adapter-dimer peak or substantial fragments outside the 200–700 bp region are detected, perform an additional SPRIselect cleanup or size-selection step, as appropriate, following the manufacturer’s instructions (Figure 3B).
  d. After cleanup or size selection, reassess the library using the TapeStation and recalculate the molar concentration within the 200–700 bp region before pooling.

***Note:*** As a practical target, aim for a region molarity of approximately 2 nM or higher per library to permit normalization and equimolar pooling. Do not treat this value as a strict acceptance threshold because the required concentration depends on the sequencing platform, the number of libraries in the pool, and the requirements of the sequencing facility. In the example dataset, a representative library with a region molarity of approximately 7.8 nM within the 200–700 bp region was successfully sequenced.

**CRITICAL:** Additional cleanup or size selection can reduce library recovery and may also remove some target library fragments. Balance removal of unwanted fragments against preservation of the desired library.

### Library pooling and sequencing of CUT&RUN libraries

**Timing: ∼1 h, plus sequencing time**

This step pools quality-controlled CUT&RUN libraries and prepares them for sequencing on an Illumina platform. Library pooling should be based on the TapeStation results obtained in Step 69.

70. Pool the libraries equimolarly based on the molar concentrations calculated for the 200–700 bp region in Step 69.
71. Sequence the pooled libraries on an appropriate Illumina platform based on the target sequencing depth and number of libraries.

***Note:*** Include an IgG negative-control library when available to assess background and specificity. Ideally, prepare a stage-matched IgG control for each developmental stage. In the example dataset, an E11.5 wild-type IgG library was available, whereas no stage-matched IgG library was available for E13.5. The IgG control was used for background and specificity assessment and was not used to determine the threshold for final peak calling (see Step 99).

***Note:*** For accurate quantification of the pooled library prior to sequencing, qPCR-based methods (e.g., KAPA Library Quantification Kit) are recommended. While TapeStation provides an estimate of molar concentration, qPCR specifically measures only library molecules with both adapters and is more accurate for determining the loading concentration on sequencers.

**Pause point:** Purified CUT&RUN libraries can be stored at −20°C for up to 4 weeks before sequencing. Follow the storage instructions provided by your sequencing facility and library preparation kit.

### Embryo collection and forelimb bud dissection for scRNA-seq

**Timing: 2–4 h (depending on the number of embryos recovered)**

This step describes the collection of embryos and dissection of forelimb buds for downstream scRNA-seq analysis. Rapid dissection under cold conditions is important for preserving cell viability and RNA quality.

72. Prepare wild-type ICR pregnant mice that have reached the desired developmental stage for scRNA-seq analysis.
73. Follow the embryo collection and forelimb bud dissection procedure described for CUT&RUN samples in Steps 2–5, with the following modifications:
  a. Do not collect the amniotic membrane for genotyping.
  b. Collect the dissected forelimb buds into 1.5 mL tubes labeled for scRNA-seq (Figure 1).
74. Immediately proceed to the single-cell suspension preparation step with the collected forelimb buds.

**CRITICAL:** Perform all operations from embryo collection through forelimb bud dissection rapidly under cold conditions to minimize loss of cell viability and RNA quality.

**CRITICAL:** Apply consistent criteria for developmental stage determination and forelimb bud excision boundaries across all samples. Variability in these factors can affect cell composition and gene expression profiles.

### Preparation of single-cell suspensions from embryonic forelimb buds for scRNA-seq

**Timing: 1.5–2.5 h**

This step generates single-cell suspensions from embryonic forelimb buds for downstream scRNA-seq library preparation. Cell dissociation is performed similarly to the CUT&RUN workflow, except that fixation-related steps are omitted.

75. Process the scRNA-seq forelimb buds following the cell dissociation procedure described for CUT&RUN samples (Steps 7–24), with the following modifications:
  a. Omit the fixation (Step 9), glycine quench (Step 11), and post-fixation wash steps (Steps 12–13). Proceed directly from tissue mincing (Step 7) to collagenase D treatment (Steps 14–16).
  b. After dissociation and the final washing steps, resuspend the cell pellet in HBSS to obtain a single-cell suspension.

***Note:*** During collagenase D treatment, assess the extent of tissue dissociation every 30 min when gently tapping the samples, as described in Step 15a. Unfixed tissues used for scRNA-seq generally dissociate more rapidly than fixed tissues used for CUT&RUN therefore, terminate the collagenase D treatment once sufficient dissociation is achieved.

76. Measure the cell concentration and viability using an automated cell counter, and assess the extent of cell aggregation.
  a. Record the percentage of viable cells.
    **CRITICAL:** Do not proceed directly to scRNA-seq library preparation if cell viability is below 70%. Follow the options described in Step 76d.
    ***Note:*** A viability of 80%–95% is optimal for single-cell library preparation. Cells damaged during dissociation release free RNA and nucleases into the suspension, increasing ambient RNA background and the proportion of empty droplets in the sequencing data.
  b. Assess cell aggregation.
    i. Inspect the brightfield counting image and cell-size distribution to confirm that the suspension consists predominantly of single cells.
    ii. If visible clumps are present, or if a pronounced shoulder or secondary population appears at larger sizes, gently resuspend the cells by pipetting and reassess the suspension.
    iii. If clumps persist, pass the suspension through a cell strainer with a smaller pore size than the 70 μm strainer used during dissociation in Step 17, and reassess the suspension.
      ***Note:*** The automated cell counter used in this protocol reports cell concentration, viability, and size distribution but does not provide a numerical aggregation rate. A narrow size distribution centered on the expected single-cell diameter, with few events at two or more times that diameter, indicates limited aggregation. The mean diameter of dissociated E11.5–E13.5 forelimb bud cells is approximately 5 μm (Figure 1E).
  c. Retain the raw counting output for every sample.
    i. Save at least the brightfield counting image and numerical cell-count report.
      ***Optional:*** If a fluorescence-capable cell counter is available, stain the cells with acridine orange/propidium iodide (AO/PI) and retain the FL1 live-cell and FL2 dead-cell channel plots in addition to the brightfield image.
      ***Note:*** In this protocol, cell concentration and viability were assessed using a Countess II FL in brightfield mode with trypan blue exclusion; the fluorescence channels were not used. AO/PI-based fluorescence measurements may provide a more reliable viability estimate for dissociated embryonic tissue, particularly when debris is abundant.
  d. Address cell viability below 70% before library preparation.
    i. Re-prepare the sample using optimized dissociation conditions or perform dead-cell removal using a magnetic bead–based or column-based method, and then reassess viability.
    ii. After dead-cell removal, confirm that sufficient cells remain for the intended number of reactions. For low-input samples such as E11.5 forelimb buds, verify the recovered cell number before beginning library preparation.
      ***Note:*** Dead-cell removal can result in substantial cell loss and was not performed in the example dataset.
      **CRITICAL:** Assess viability immediately before library preparation. The viability of dissociated embryonic cells declines rapidly, and measurements obtained substantially earlier may overestimate the quality of the suspension that is ultimately loaded.
      **CRITICAL:** Do not fix cells intended for scRNA-seq. Trypan blue enters cells with permeabilized membranes, causing fixed cells to be scored as dead and preventing reliable viability assessment (Figure 1E).

### Single-cell library preparation and sequencing

**Timing: ∼6–8 h, plus sequencing time**

This step converts dissociated forelimb bud cells into sequencing-ready scRNA-seq libraries using the 10x Genomics Chromium platform. Library preparation follows the manufacturer’s protocols with Cell Multiplexing and dual-index chemistry.

77. Label cells from each developmental stage with Cell Multiplexing Oligos (CMOs) using the 10x Genomics 3′ CellPlex Kit Set A. Pool the labeled cells prior to loading onto the Chromium platform.

***Note:*** In this protocol, cells from E9.5 and E10.5 were labeled with CMO304 and CMO305, respectively, and pooled. Cells from E11.5, E12.5, and E13.5 were labeled with CMO301, CMO302, and CMO303, respectively, and pooled.

78. Prepare gene expression and Feature Barcode libraries using the Chromium Next GEM Single Cell 3′ Reagent Kits v3.1 (Dual Index) according to the manufacturer’s instructions (https://www.10xgenomics.com/support/single-cell-gene-expression/documentation).
79. Sequence the pooled libraries by paired-end sequencing on an Illumina NovaSeq X Plus platform.

**CRITICAL:** Single-cell suspension quality directly affects the final data quality. Minimize contamination by dead cells, debris, and cell clumps, and proceed to GEM generation as quickly as possible after cell preparation.

**CRITICAL:** Reaction conditions, cell input numbers, and library preparation parameters depend on the kit version and platform used. Always follow the specific 10x Genomics protocol for the kit version employed.

**Pause point:** Completed scRNA-seq libraries can be stored at −20°C for up to 4 weeks before sequencing. Follow the storage instructions provided by the library kit and sequencing facility.

### Preprocessing and clustering of scRNA-seq data

**Timing: 0.5–1 day (depending on dataset size and computing environment)**

This section describes the processing of raw scRNA-seq data to generate clustered and annotated single-cell transcriptional profiles. The resulting cluster-specific marker genes and differentially expressed genes are used for downstream integration with CUT&RUN data.

80. If starting from BCL files, demultiplex the raw sequencing data using BCL Convert or cellranger mkfastq to generate FASTQ files. Skip this step if FASTQ files are provided by the sequencing facility.
81. Process the gene expression and Cell Multiplexing Oligo (CMO) library FASTQ files using the cellranger multi pipeline in Cell Ranger v8.0.1, with GRCm39 as the mouse reference genome, to generate stage-specific gene–cell count matrices.
82. Import the resulting stage-specific gene–cell count matrices into Seurat v5.1.0 in R v4.4.0 for downstream analysis.
83. Calculate quality-control metrics for each cell, including the number of detected genes, UMI counts, and the percentage of mitochondrial transcripts. Inspect the distributions of these metrics using violin plots or similar visualizations.
84. Exclude low-quality cells and obvious outliers.
  a. Exclude cells with fewer than 200 or more than 12,000 detected genes, or with more than 5% mitochondrial transcripts.

***Note:*** Dedicated doublet-detection tools, such as DoubletFinder or scDblFinder, were not applied to the example dataset. However, we recommend their use, particularly when the aggregation rate of the input cell suspension is unknown or exceeds the recommended threshold described in Step 76.

**CRITICAL:** QC-filtering thresholds may vary depending on developmental stage, sample quality, and cell-recovery efficiency. Although the thresholds described above were used in this study, determine appropriate thresholds only after examining the distribution of each metric for each sample, for example using violin plots.

85. Normalize the filtered data using NormalizeData() in Seurat with normalization.method = "LogNormalize" and scale.factor = 10,000, and identify 2,000 highly variable genes using FindVariableFeatures() with selection.method = "vst".
86. Scale the data using the highly variable genes and perform principal component analysis (PCA).
87. Select principal components for downstream analysis and construct a nearest-neighbor graph.
  a. Inspect a PCA elbow plot or a similar diagnostic when selecting principal components.

***Note:*** In this study, all 50 principal components calculated by PCA (PCs 1–50) were used for downstream nearest-neighbor graph construction, UMAP visualization, and clustering.

88. Perform clustering.
  a. Set the clustering resolution to 0.8.
  b. Visualize the cells using UMAP.
89. Identify marker genes for each cluster and annotate cell types or cell states based on the expression of known developmental marker genes.
90. Compare cell composition across developmental stages and identify Sox9-high clusters for downstream analysis.
  a. Compare cell composition and cluster distribution across developmental stages.
  b. Visualize Sox9 expression in each cluster using FeaturePlot or VlnPlot in Seurat.
  c. Calculate the mean Sox9 expression level for each cluster and inspect its distribution across clusters (Figure 4B).

**Figure 4:**
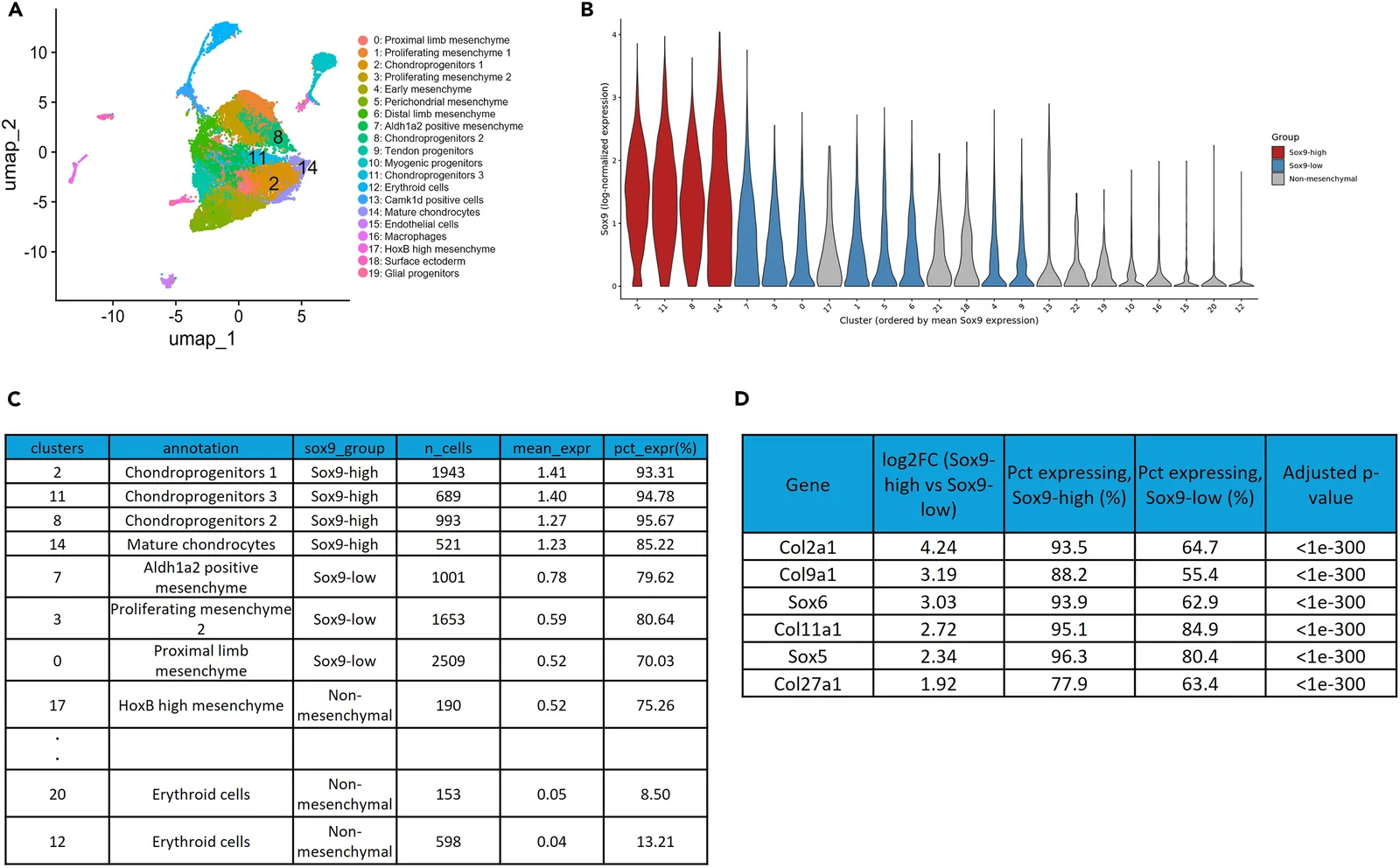
Classification of cell clusters based on *Sox9* expression and characterization of Sox9-high cell populations (A) UMAP plot showing cell clusters identified from the scRNA-seq dataset. Colors indicate annotated cell types, and numbers indicate cluster identities. (B) Violin plot showing the log-normalized expression of *Sox9* in each cluster. Clusters are ordered according to their mean *Sox9* expression levels. Based on *Sox9* expression and cell-type annotations, clusters were classified as Sox9-high (red), Sox9-low (blue), or non-mesenchymal (gray). (C) Summary of the cell-type annotation, *Sox9* expression group, number of cells (n_cells), mean *Sox9* expression (mean_expr), and percentage of *Sox9*-expressing cells (pct_expr) for each cluster. The Sox9-high group includes chondroprogenitor and mature chondrocyte clusters with high *Sox9* expression. (D) Expression differences in representative cartilage-related genes between the Sox9-high and Sox9-low groups. For each gene, the log2 fold change (log2FC) in the Sox9-high group relative to the Sox9-low group and the percentage of expressing cells in each group are shown. The scRNA-seq data were reanalyzed from Sega et al.^1^ Panel (A) was adapted from Figure 2A of Sega et al.^1^ under the Creative Commons Attribution 4.0 International (CC BY 4.0) license.
  d. Where a clear separation is evident, designate the clusters above the separation as Sox9-high clusters.
  e. Confirm the classification using established markers of the corresponding lineage.
  f. Select the confirmed Sox9-high clusters for downstream differential expression analysis and integration with CUT&RUN data.

***Note:*** In the example dataset, four clusters, chondroprogenitors 1–3 and mature chondrocytes, showed mean *Sox9* expression values of 1.23–1.41, whereas the next-highest cluster had a mean value of 0.78 (Figure 4C). These four clusters were classified as Sox9-high, and the remaining eight mesenchymal clusters as Sox9-low. Non-mesenchymal clusters were excluded. The classification was supported by higher expression of established chondrogenic genes, including *Col2a1, Col9a1, Col11a1, Col27a1, Sox5,* and *Sox6,* in the Sox9-high clusters (Figure 4D).

91. Perform two distinct differential expression analyses using FindMarkers in Seurat.
  a. Use the Wilcoxon rank-sum test.
  b. Define genes as differentially expressed when the adjusted p value is below 0.01 and the absolute log_2_ fold change is greater than 1.
  c. Perform both analyses separately for each Sox9-high cluster at each developmental stage.
    i. Pan-chondrogenic DEGs: Compare each Sox9-high cluster with the combined Sox9-low mesenchymal clusters.
    ii. Cluster-specific DEGs: Compare each Sox9-high cluster with the combined population of the remaining Sox9-high clusters.
  d. Confirm that each comparison group contains at least 10 cells. Exclude and record any cluster–stage combination that does not meet this minimum.
  e. Save both DEG sets for every cluster–stage combination for subsequent integration with the CUT&RUN data.
92. From each DEG set generated in Step 91, retain only genes upregulated in the Sox9-high cluster (adjusted p < 0.01 and log_2_FC > 1). Keep the pan-chondrogenic and cluster-specific DEG sets separate for downstream integration.

Run the following example R code for Steps 91–92 (pan-chondrogenic comparison shown):

deg <- FindMarkers(

 obj,

 ident.1 = high_cells,

 ident.2 = low_cells,

 test.use = "wilcox",

 logfc.threshold = 0.1,

 min.pct = 0.01

)

deg_up <- deg |>

 tibble::rownames_to_column("gene") |>

 dplyr::filter(p_val_adj < 0.01, avg_log2FC > 1) |>

 dplyr::arrange(dplyr::desc(avg_log2FC))

write.csv(deg_up, "<stage>_cluster<N>_DEG.csv", row.names = FALSE)

***Note:*** The logfc.threshold = 0.1 setting is used only as an initial filter in FindMarkers; the final criterion for upregulated DEGs used in downstream integration is avg_log2FC > 1 with an adjusted p value < 0.01.

### Preprocessing and quality assessment of CUT&RUN data

**Timing: 0.5–1 day (depending on dataset size and computing environment)**

This major step processes CUT&RUN sequencing data and evaluates library quality before peak calling. Apply the same alignment, filtering, and normalization parameters to all samples.

93. Assess and preprocess the raw sequencing reads.
  a. Assess the quality of the raw sequencing data using FastQC v0.12.1.
  b. Remove adapter sequences and low-quality bases using Trim Galore v0.6.10.
  c. Reassess read quality using FastQC after trimming.
94. Align the quality-controlled reads to the mouse reference genome and generate sorted and indexed BAM files.
  a. Construct the Bowtie2 index from the GENCODE release M33 primary assembly genome FASTA for GRCm39.
  b. Align the quality-controlled reads to the mouse reference genome using Bowtie2 v2.5.4 with the following command:
    bowtie2 --end-to-end --very-sensitive --no-mixed --no-discordant
    -I 10 -X 700 -p <threads> ∖
    -x <mouse_index> ∖
    -1 <R1.fastq.gz> -2 <R2.fastq.gz> ∖
    -S <sample>.sam
  c. Use SAMtools v1.22.1 to convert the resulting SAM files into sorted and indexed BAM files.
    **CRITICAL:** Construct the Bowtie2 index from the same genome release corresponding to the GTF file used for peak annotation in Step 100. Use of the primary assembly avoids multimapping ambiguity caused by alternative haplotype sequences and patch scaffolds included in some full assembly FASTA files.
95. Align the same quality-controlled reads to the E. coli genome using Bowtie2.
  a. Record the alignment rate and read count for each library.
    ***Note:*** In this protocol, use a Bowtie2 index constructed from the *E. coli* K-12 substr. MG1655 reference genome (RefSeq assembly GCF_000005845.2, ASM584v2).
  b. Perform the mouse and *E. coli* alignments independently rather than using a concatenated mouse–*E. coli* reference genome.
    ***Note:*** Because the mouse and *E. coli* alignments are performed independently using the same reads, the resulting alignment fractions are not strictly mutually exclusive.
    ***Note:*** Because spike-in DNA is added after MNase digestion, spike-in normalization does not correct for differences in input cell number or CUT&RUN reaction efficiency.
96. Remove PCR duplicates and assess the fragment-length distribution.
  a. Use MarkDuplicates in Picard v3.4.0 with REMOVE_DUPLICATES=true to generate filtered BAM files.
  b. Use CollectInsertSizeMetrics in Picard v3.4.0 to obtain the fragment-length distribution.

***Note:*** In the example dataset, PCR duplicate removal was the only explicit read-filtering step. Low-MAPQ and mitochondrial reads were retained.

**CRITICAL:** Apply identical filtering criteria to all samples. Otherwise, differences in peak number or signal intensity may reflect differences in data processing rather than biological variation.

97. Generate spike-in-normalized bedGraph and BigWig files for peak calling and coverage-track visualization.
  a. Calculate the scale factor for each library using the *E. coli* alignment generated in Step 95.
    $$\text{scale}\;\text{factor}=\frac{10,000}{\text{number}\;\text{of}\;\text{fragments}\;\text{mapped}\;\text{to}\;E.\;coli}$$
    Count read 1 records to determine the number of mapped fragments.
    samtools view -c -f 64 <E_coli_mapped>.bam
  b. Record each calculated value together with the corresponding library identifier.
    ***Note:*** The spike-in BAM files used here contain only mapped records because unmapped reads were removed upstream using samtools view -F 0x04; flagstat consequently reports a mapping rate of 100%. Because alignment was performed using the --no-mixed and --no-discordant options, all aligned read pairs in these BAM files are concordant, and the number of read 1 records corresponds to the number of fragments. The numerator of 10,000 is an arbitrary constant applied identically to all libraries to keep the resulting scale factors within a convenient numerical range.
  c. Convert the filtered BAM file generated in Step 96 into a fragment-level BED file.
    samtools sort -n <filtered>.bam -o <sample>.namesorted.bam
    bedtools bamtobed -bedpe -i <sample>.namesorted.bam |∖
    awk '$1==$4 && $6-$2 < 1000' |
    cut -f 1,2,6 |∖
    sort -k1,1 -k2,2n -k3,3n > <sample>.fragments.bed
    ***Note:*** bedtools bamtobed -bedpe outputs paired-end BED records while retaining mate information and therefore requires a BAM file sorted by read name using samtools sort -n. The subsequent awk command retains pairs mapped to the same chromosome with fragment lengths of less than 1,000 bp. The cut -f 1,2,6 command then generates a BED file in which each interval represents the complete fragment spanning the two aligned mates.
  d. Apply the scale factor calculated in Step 97a to generate the normalized bedGraph file.
    bedtools genomecov -bg -scale <scale_factor>∖
     -i <sample>.fragments.bed∖
     -g genome.chrom.sizes∖
     > <sample>.spikein_norm.bedgraph
  e. Generate the corresponding BigWig file for visualization using bamCoverage from deepTools v3.5.5. Apply the same scale factor using the --scaleFactor option. Use the same normalization factor for the bedGraph and BigWig files, and apply the same procedure to all libraries.
    **CRITICAL:** The bedGraph files generated here have already been normalized using spike-in-derived scale factors. When performing peak calling with SEACR, specify non as the third argument to disable control normalization (Step 99). Combining spike-in scaling with the SEACR norm option would apply normalization twice.
    **CRITICAL:** Because spike-in DNA is added after MNase digestion, spike-in normalization does not correct for differences in CUT&RUN reaction efficiency. Therefore, normalized signal intensities should be interpreted cautiously when comparing samples quantitatively.
    ***Note:*** Fragment coordinates are generated directly from the Bowtie2 and SAMtools outputs. The only information derived from the spike-in alignment is the scale factor applied to the signal values. Scaling therefore changes signal height but does not alter fragment or peak coordinates.
98. Evaluate CUT&RUN library quality before peak calling using the approximate read composition, fragment-length distribution, number of unique read pairs after duplicate removal.
  a. Summarize the approximate read composition as the fractions aligned independently to the mouse genome, aligned independently to the *E. coli* spike-in genome, and remaining after subtraction.
    ***Note:*** The remaining fraction includes adapter dimers, low-quality reads, and other contaminants. In the example dataset, the approximate mouse-aligned, *E. coli*-aligned, and remaining fractions were 70.3%, 15.5%, and 14.2%, respectively, at E11.5 and 50.7%, 7.8%, and 41.6%, respectively, at E13.5 (Figure 5A). A high spike-in fraction indicates relatively low endogenous chromatin recovery, whereas a high remaining fraction may indicate library-preparation or sequencing-quality issues.
    **Figure 5:**
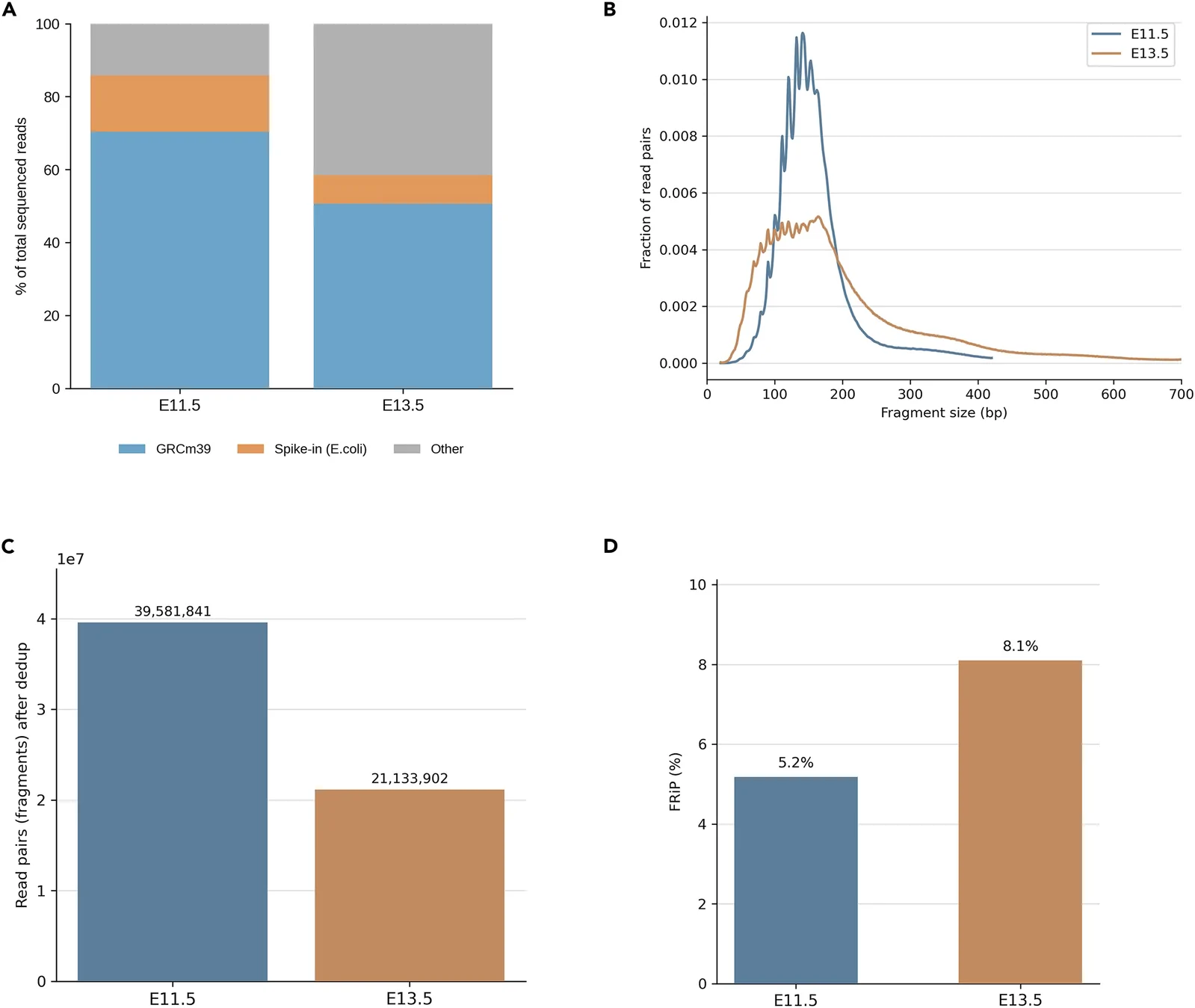
Quality control of CUT&RUN sequencing data at E11.5 and E13.5 (A) Distribution of sequenced reads mapped to the mouse reference genome (GRCm39), *E. coli* spike-in DNA, or other sequences for the E11.5 and E13.5 CUT&RUN datasets. Values are shown as percentages of total sequenced reads. (B) Fragment size distributions of paired-end reads in the E11.5 and E13.5 CUT&RUN datasets. The x-axis indicates fragment size in base pairs, and the y-axis indicates the fraction of read pairs. (C) Number of read pairs remaining after deduplication for the E11.5 and E13.5 CUT&RUN datasets. Values above the bars indicate the number of deduplicated read pairs. (D) Fraction of reads in peaks (FRiP) for the E11.5 and E13.5 CUT&RUN datasets. Values above the bars indicate the FRiP percentage. The CUT&RUN data were reanalyzed from Sega et al.^1^
  b. Examine the fragment-length distribution.
    ***Note:*** CUT&RUN targeting a transcription factor is expected to produce a distribution dominated by fragments shorter than approximately 200 bp. A broad distribution with a pronounced tail extending beyond 300 bp suggests over-fixation, insufficient digestion, or both. Although the E11.5 and E13.5 libraries in the example dataset were prepared using the same conditions, the E13.5 profile was substantially broader (Figure 5B). Tissue mass and cell number increase considerably between these developmental stages; therefore, conditions optimized for one stage may not be directly applicable to another.
  c. Determine the number of unique read pairs remaining after duplicate removal.
    ***Note:*** The example dataset yielded 3.96 × 10^7^ unique read pairs at E11.5 and 2.11 × 10^7^ at E13.5 (Figure 5C). As a practical target for this workflow, aim for approximately 1 × 10^7^ or more unique read pairs per library. A high duplicate rate alone does not indicate library failure; however, a low number of unique reads after duplicate removal indicates limited library complexity.
    ***Note:*** Do not evaluate library quality based on the mouse alignment rate alone. In the example dataset, the E13.5 library had a lower mouse alignment rate but retained 2.11 × 10^7^ unique read pairs and produced a usable peak set.

### CUT&RUN peak calling and generation of promoter-associated gene lists

**Timing: 1–3 h**

This major step calls and evaluates Sox9 CUT&RUN peaks and generates promoter-associated gene lists for integration with scRNA-seq data. Apply the same peak-calling and annotation parameters to all samples.

99. Call peaks using SEACR v1.3 and evaluate the resulting peak sets.
  a. Call peaks using a numerical threshold.
    bash SEACR_1.3.sh <target>.spikein_norm.bedgraph ∖
     0.001 non stringent <output_prefix>
    ***Note:*** A numerical threshold of 0.001 retains the top 0.1% of signal blocks ranked by area under the curve (AUC). In the example dataset, this procedure produced 3,584 peaks at E11.5 and 4,345 peaks at E13.5.
    **CRITICAL:** The input bedGraph files have already been normalized using spike-in-derived scale factors. Specify non as the third argument to disable control normalization and avoid applying normalization twice. Do not use the IgG control library to determine the threshold for the final peak sets.
  b. Calculate FRiP using the peak set called in Step 99a. Intersect the peak intervals with the fragment BED file and calculate the fraction of fragments that overlap at least one peak.
    ***Note:*** FRiP depends on the peak-calling threshold and should therefore be interpreted together with the peak caller and parameters used. The FRiP values for the example dataset were 5.2% at E11.5 and 8.1% at E13.5 (Figure 5D).
  c. Inspect genome-browser coverage tracks at known target loci and compare the target and IgG tracks at the same genomic regions. For Sox9, inspect the *Col11a1* and *Col27a1* loci (Figure 6D).
    ***Note:*** A stage-matched E11.5 WT IgG library was available for the E11.5 target library. Because a stage-matched IgG library was not available for E13.5, use the E11.5 WT IgG track only as an exploratory reference when inspecting the E13.5 target track.
    ***Note:*** When available, compare CUT&RUN peaks with published datasets for the same factor and with relevant chromatin marks, such as H3K27ac, as orthogonal support for peak specificity, particularly when biological replicates are unavailable.
    ***Note:*** The FRiP values calculated using the numerical-threshold peak sets were 5.2% and 8.1% at E11.5 and E13.5, respectively, whereas the control-based peak sets produced FRiP values of 21.0% and 13.2%. A FRiP exceeding 20% for a peak set containing more than 10^5^ peaks is more likely to reflect broad genomic coverage by the peak set than genuine concentration of signal within specific regulatory regions.
    **CRITICAL:** Control-based peak calling using the E11.5 WT IgG library was explored but was not used to define the final peak sets. It yielded substantially larger peak sets than numerical thresholding (133,778 vs. 3,584 peaks at E11.5 and 75,367 vs. 4,345 peaks at E13.5), and the E11.5 IgG library was not stage matched to E13.5. Therefore, the numerical threshold of 0.001 was used consistently for final peak calling at both developmental stages.
    ***Optional:*** When at least two independent biological replicates are available, assess peak reproducibility as follows:

**Figure 6:**
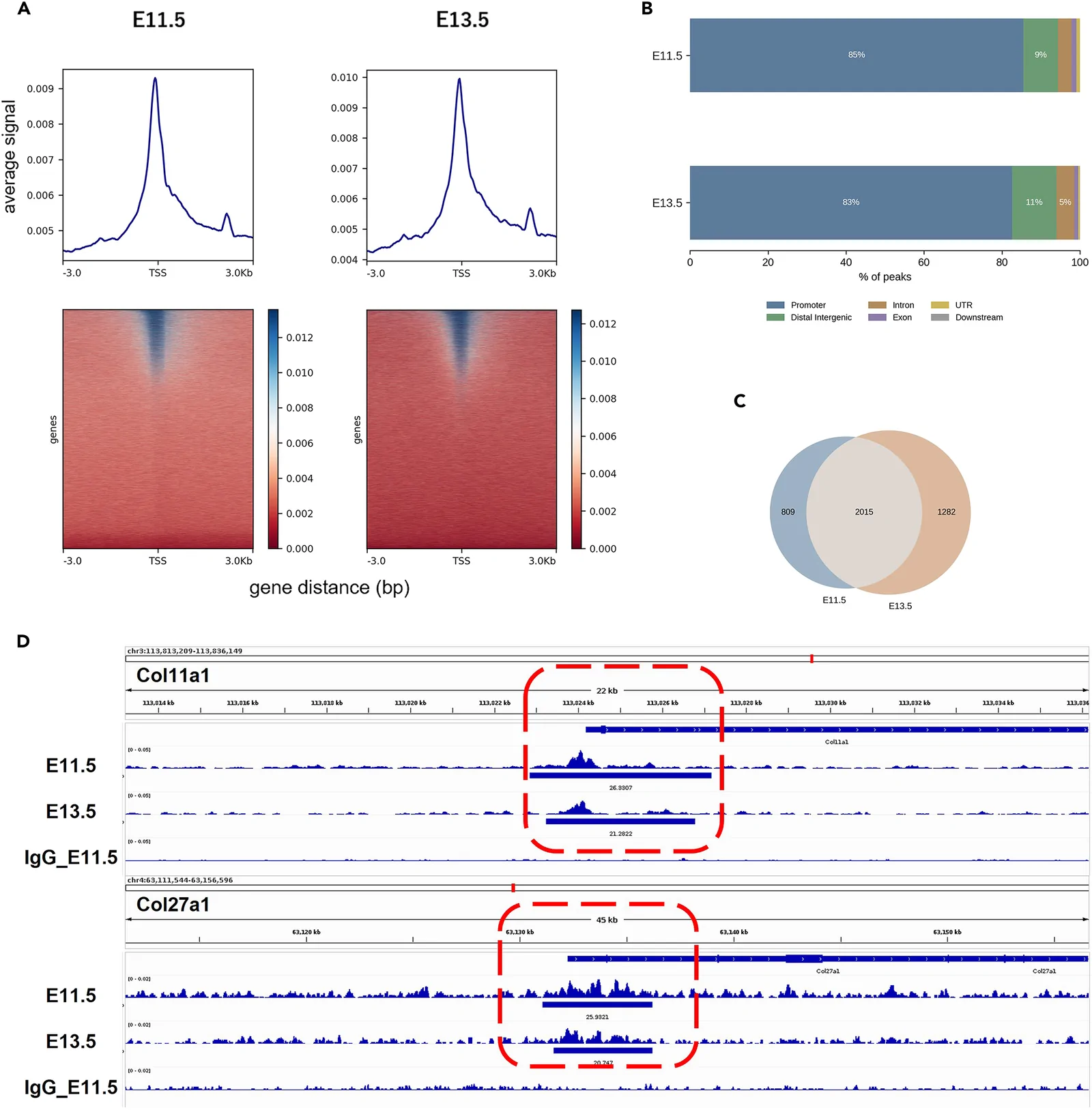
Genomic distribution of Sox9 CUT&RUN peaks and signals at representative target loci at E11.5 and E13.5 (A) Average signal profiles and heatmaps of Sox9 CUT&RUN signals around transcription start sites (TSSs) at E11.5 and E13.5. CUT&RUN signals are shown within ±3 kb of the TSS. (B) Genomic annotation of Sox9 CUT&RUN peaks identified at E11.5 and E13.5 using SEACR (top 0.001). Peaks were classified into promoter, distal intergenic, intronic, exonic, UTR, or downstream regions, and the proportion of peaks in each category is shown. (C) Venn diagram showing the overlap between promoter-associated genes identified from the E11.5 and E13.5 Sox9 CUT&RUN peak sets. The numbers of stage-specific and shared genes are indicated. (D) Genome browser tracks showing Sox9 CUT&RUN signals at the representative cartilage-related genes *Col11a1* and *Col27a1*. Sox9 CUT&RUN signals at E11.5 and E13.5, together with the E11.5 IgG control, are shown. Red dashed boxes indicate representative regions showing Sox9 binding signals.
  d. Assess peak overlap between replicates using, for example, bedtools intersect or the Jaccard statistic.
  e. Define a consensus peak set comprising regions reproducibly detected in multiple replicates.
  f. Use the consensus peak set for downstream annotation and integration.
    ***Note:*** The example dataset contains one library per developmental stage; therefore, individual stage-specific peak sets were used for downstream analysis.
100. Annotate the CUT&RUN peaks and assign them to genes.
  a. Generate a TxDb object from the GENCODE vM33 annotation GTF for GRCm39 using txdbmaker::makeTxDbFromGFF() from txdbmaker v1.6.2.
  b. Annotate the peaks using ChIPseeker v1.46.1 and the TxDb object generated in substep a.
  c. Define promoter regions as TSS ± 3 kb and assign each peak to its nearest gene.
  d. Use org.Mm.eg.db v3.22.0 for gene-symbol and Entrez ID mapping.

library(txdbmaker)

library(ChIPseeker)

txdb <- makeTxDbFromGFF(

 "gencode.vM33.annotation.gtf",

 format = "gtf"

)

peakAnno <- annotatePeak(

 peaks,

 TxDb = txdb,

 tssRegion = c(-3000, 3000),

 annoDb = "org.Mm.eg.db"

)

***Note:*** Of the 3,584 E11.5 and 4,345 E13.5 SEACR peaks, 31 and 24 peaks, respectively, were located on scaffolds not represented in the GENCODE vM33-derived TxDb and were excluded. Thus, 3,553 E11.5 and 4,321 E13.5 peaks were retained for downstream annotation.

**CRITICAL:** Use the same genome assembly and gene-annotation release throughout the workflow. In this protocol, reads are aligned to GRCm39, and GENCODE vM33 is used for gene annotation in both CUT&RUN and scRNA-seq analyses.

101. Generate a promoter-associated gene table for integration with the scRNA-seq dataset.
  a. Retain peaks annotated by ChIPseeker as Promoter (<=1kb), Promoter (1-2kb), or Promoter (2-3kb), which collectively correspond to TSS ± 3 kb.
  b. Collapse multiple promoter-associated peaks assigned to the same gene into a single gene-level record.
  c. Use the values in column V4 of the imported SEACR output as the peak AUC and those in column V5 as the maximum signal.

Run the following R code:

anno_df <- as.data.frame(peakAnno)

promoter_gene_table <- anno_df |>

 dplyr::filter(

 grepl("ˆPromoter", annotation),

 !is.na(SYMBOL)

 ) |>

 dplyr::group_by(SYMBOL) |>

 dplyr::summarise(

 n_peaks = dplyr::n(),

 max_AUC = max(V4),

 max_signal = max(V5),

 nearest_peak_distance_to_TSS =

 distanceToTSS[which.min(abs(distanceToTSS))],

 peak_coords = paste0(

 seqnames, ":", start, "-", end,

 collapse = ";"

 ),

 ensembl_id = paste(

 unique(geneId),

 collapse = ";"

 ),

 .groups = "drop"

 ) |>

 dplyr::arrange(dplyr::desc(max_AUC))

write.csv(

 promoter_gene_table,

 "<sample>_promoter_peak_genes.csv",

 row.names = FALSE

)

***Note:*** The output columns are SYMBOL, n_peaks, max_AUC, max_signal, nearest_peak_distance_to_TSS, peak_coords, andensembl_id. The nearest_peak_distance_to_TSS value is the signed distance of the promoter-associated peak closest to the TSS. The numbers of peaks and genes retained at each filtering step are summarized in Table 1.

***Note:*** In the example dataset, 3,037 of 3,553 annotated E11.5 peaks (85.5%) and 3,569 of 4,321 annotated E13.5 peaks (82.6%) were promoter-proximal. After collapsing multiple peaks associated with the same gene, the resulting tables contained 2,824 promoter-associated genes at E11.5 and 3,297 at E13.5.

***Note:*** Use only genes associated with promoter-proximal peaks for the integration analysis. Confidence in simple nearest-gene assignment generally decreases as the distance from the TSS increases. Because promoter-proximal peaks account for most of the annotated peaks in the example dataset, this restriction removes a relatively small fraction of peaks.

**CRITICAL:** Restricting the integration to promoter-proximal peaks excludes distal regulatory elements. If distal peaks are included, use an appropriate strategy to evaluate enhancer–gene relationships and clearly report the peak-to-gene assignment method.

***Optional:*** Perform motif-enrichment analysis using HOMER v5.1 to identify sequence motifs enriched within Sox9-bound regions.

**CRITICAL:** Peak-calling results depend strongly on the handling of duplicate reads, filtering criteria, and peak-calling parameters. Apply the same analysis settings to all samples.

**Table 1:** Number of peaks and genes retained at each filtering step in the example dataset

Stage	Peaks called (SEACR)	Peaks annotated	Promoter-proximal peaks	Peaks with an assigned gene symbol	Genes after collapsing
E11.5	3584	3553	3037 (85.5%)	2873	2824
E13.5	4345	4321	3569 (82.6%)	3359	3297

### Integration of scRNA-seq and CUT&RUN datasets

**Timing: 2–6 h**

This major step integrates the DEG sets generated in Step 92 with the promoter-associated gene sets generated in Step 101 to identify candidate Sox9 target genes in embryonic forelimb buds. Because the two datasets were generated using different experimental workflows, carefully harmonize gene annotations and interpret the results separately for each developmental stage.

102. Intersect the scRNA-seq DEG sets with the promoter-associated gene sets to define candidate Sox9 target genes. For each developmental stage represented in both datasets, separately intersect each DEG set with the promoter-associated gene set from the corresponding developmental stage.
  a. Intersect the pan-chondrogenic DEGs with the promoter-associated genes.
  b. Intersect the cluster-specific DEGs with the promoter-associated genes.

Run the following R code.

peak_genes <- read.csv("<sample>_promoter_peak_genes.csv")

candidates <- deg_up |>

 dplyr::filter(gene %in% peak_genes$SYMBOL) |>

 dplyr::left_join(peak_genes, by = c("gene" = "SYMBOL")) |>

 dplyr::arrange(dplyr::desc(avg_log2FC))

write.csv(candidates, "<stage>_cluster<N>_candidates.csv", row.names = FALSE)

***Note:*** In the example dataset, these intersections generated stage- and cluster-resolved candidate lists at E11.5 and E13.5.

103. Prioritize the resulting candidates for experimental follow-up using the following criteria.
  a. Evaluate the maximum CUT&RUN peak AUC and signal associated with each gene (max_AUC and max_signal generated in Step 101).
  b. Consider the distance between the nearest promoter-associated peak and the TSS (nearest_peak_distance_to_TSS).
  c. Evaluate the magnitude of differential expression in the corresponding cluster.
  d. Determine whether the gene is identified in the cluster-specific or pan-chondrogenic candidate list.
  e. Assess consistency with published binding data and established lineage biology.

***Note:*** Candidate prioritization is interpretive and was not based on a single automated score or fixed scoring formula. In the example dataset, the criteria above were considered jointly, and selected candidates were further inspected using genome-browser tracks. The parameters and decision criteria used in the example dataset are summarized in Table 2.

***Note:*** Perform the intersection for every combination of DEG type, Sox9-high cluster, and developmental stage for which both data types are available. In the example dataset, two DEG types × four Sox9-high clusters × two developmental stages yielded 16 candidate lists.

**CRITICAL:** Before intersection, verify that gene identifiers are consistent between the scRNA-seq and CUT&RUN datasets and document genes present in only one dataset.

**CRITICAL:** Overlap between an upregulated DEG and a promoter-associated CUT&RUN gene identifies a strong candidate for Sox9 regulation but does not establish functional causality. Interpret each candidate in the context of developmental stage, cell population, peak position, and independent biological evidence.

**Table 2:** Parameters and decision criteria used to generate the candidate Sox9 target gene lists in the example dataset

Workflow stage	Parameter	Setting used in the example dataset	Step
scRNA-seq processing	Reference	GRCm39 (Cell Ranger reference)	81
	Cell filtering	200–12000 detected genes; <5% mitochondrial transcripts	84
	Doublet detection	Not applied; upper gene-count limit used instead	84
	Principal components	PCs 1–50	87
	Clustering resolution	0.8	88
Definition of Sox9-high clusters	Criterion	Mean Sox9 expression per cluster; group boundary placed at the largest gap in the distribution	90c-d
	Sox9-high	Four clusters, mean expression 1.23–1.41 (chondroprogenitors 1–3, mature chondrocytes)	90d
	Sox9-low	Eight mesenchymal clusters, mean expression 0.35–0.78	90
	Excluded	All non-mesenchymal clusters	90
	Validation	Enrichment of Col2a1, Col9a1, Col11a1, Col27a1, Sox5, and Sox6 in Sox9-high vs Sox9-low clusters	90e
Differential expression	Test	Wilcoxon rank-sum (FindMarkers, Seurat v5.1.0)	91
	DEG definition	Adjusted p < 0.01 and |log2FC| > 1	91
	Stratification	Performed separately for each Sox9-high cluster at each developmental stage	91
	Comparison A (pan-chondrogenic)	Sox9-high cluster vs pooled Sox9-low mesenchymal clusters	91c
	Comparison B (cluster-specific)	Sox9-high cluster vs pooled remaining Sox9-high clusters	91c
	Minimum cells per group	10	91
	Excluded combinations	Chondroprogenitor 3 at E9.5 (cluster absent, n = 0); mature chondrocytes at E9.5 (n = 4, below the minimum)	91
	Direction used for integration	Upregulated only (log2FC > 1)	92
CUT&RUN processing	Reference	GRCm39, GENCODE release M33 primary assembly	94
	Spike-in reference	E. coli K-12 substr. MG1655 (GCF_000005845.2)	95
	Read filtering	PCR duplicate removal only	96
	Normalization	Spike-in scale factor = 10000/E. coli fragment count	97a
	Fragment length filter	<1000 bp, both mates on the same chromosome	97c
Peak calling	Peak caller	SEACR v1.3	99a
	Threshold	Numeric, 0.001 (top 0.1% by AUC)	99a
	Control normalization	Disabled (non); input already spike-in normalized	99a
	Mode	stringent	99a
	Control track	Not used for threshold determination	99a
	Peaks obtained	3584 (E11.5); 4345 (E13.5)	99a
Peak annotation	Tool	ChIPseeker v1.46.1	100
	Gene models	GENCODE vM33; TxDb built with txdbmaker v1.6.2	100
	Symbol mapping	org.Mm.eg.db v3.22.0	100
	Promoter definition	TSS ±3 kb	100
	Assignment rule	Nearest gene	100
Peak set used for integration	Filter	Promoter-proximal peaks with an assigned gene symbol	101
	Genes obtained	2824 (E11.5); 3297 (E13.5); see Table1 for intermediate counts	101
Integration	Rule	Intersection of upregulated DEGs with promoter-associated genes	102
	Stage matching	DEG set and peak set from the same developmental stage	102
	Combinations	2 DEG types x 4 Sox9-high clusters x 2 stages = 16 candidate lists	102
	Stages available	E11.5 and E13.5 only (CUT&RUN not performed at E9.5, E10.5, or E12.5)	102

## Expected outcomes

Successful execution of this protocol yields integrative datasets of scRNA-seq and Sox9 CUT&RUN from mouse embryonic forelimb buds. The absolute numbers of cells, peaks, and candidate genes are specific to the example dataset and may vary depending on sample quality, library complexity, and sequencing depth. Nevertheless, comparable chondrogenic candidate Sox9 target genes may be recovered when the protocol is successfully reproduced.

From scRNA-seq, integration of data from five developmental stages (E9.5–E13.5) typically yields a single-cell dataset of approximately 20,000 cells (19,934 cells were recovered in the original study). These cells are classified into multiple clusters, and four Sox9-high populations comprising three chondroprogenitor clusters and mature chondrocytes are expected to be identified by comparing *Sox9* expression levels using violin plots (Figures 4A and 4B).

From CUT&RUN, libraries reflecting Sox9 binding regions are expected to be obtained from E11.5 and E13.5 forelimb buds. The fragment length distribution shows a peak at approximately 150 bp at both time points, and peaks are predominantly enriched within 1 kb upstream of transcription start sites (TSS) (Figures 6A and 6B). Motif analysis is expected to detect the Sox binding motif [(A/T)CAA(A/T)]. Comparison between E11.5 and E13.5 yields 2,015 shared promoter-associated genes, 809 E11.5-specific genes, and 1,282 E13.5-specific genes (Figure 6C). The original study (Sega et al.^1^) aligned CUT&RUN reads to mm10 and did not restrict the integration to promoter-proximal peaks. Gene counts obtained with this protocol therefore differ from those reported in the original publication.

Integration of these two datasets enables extraction of candidate Sox9 target genes that are highly expressed in Sox9-high clusters and harbor Sox9 binding peaks. Representative examples include *Col2a1, Col27a1, Col11a1, Col9a1, Sox5*, and *Sox6*, which are commonly detected across multiple clusters and both time points (Figure 6D). Stage-dependent candidates such as *Mbnl1* and *Wif1* (E11.5 mature chondrocytes) and *Bmp7* and *Twist2* (E13.5 mature chondrocytes) are also expected to be identified. Thus, this protocol enables inference of stage- and cluster-specific transcriptional regulation by Sox9 during development.

## Quantification and statistical analysis

All analyses were performed in R v4.4.0. For scRNA-seq, raw sequencing data were processed using Cell Ranger v8.0.1 and analyzed in Seurat v5.1.0. Cells with fewer than 200 or more than 12,000 detected genes, or with more than 5% mitochondrial transcripts, were excluded as low-quality cells. Clustering was performed with resolution 0.8, and clusters were annotated based on canonical marker genes. Differentially expressed genes (DEGs) were identified with adjusted p-value < 0.01 and absolute log2 fold change > 1 as significance thresholds.

For CUT&RUN, reads were aligned to GRCm39 (GENCODE release M33 primary assembly) using Bowtie2 v2.5.4, processed with SAMtools v1.22.1 and Picard v3.4.0, and submitted to SEACR v1.3 for peak calling with a numeric threshold of 0.001, normalization disabled (non), in stringent mode. Peaks were annotated using ChIPseeker against GENCODE vM33 gene models with a promoter definition of ±3 kb from the transcription start site. Motif enrichment was assessed using HOMER. Spike-in DNA reads were used for normalization when applicable.

For integration, candidate Sox9 target genes were defined as the intersection of promoter-associated CUT&RUN genes, defined by peaks located within ±3 kb of a transcription start site (TSS), and genes significantly upregulated in the corresponding Sox9-high cluster (adjusted *p* value < 0.01 and log_2_FC > 1). Two complementary DEG comparisons were performed: (1) each Sox9-high cluster versus the combined Sox9-low mesenchymal clusters to evaluate the general chondrogenic program and (2) each Sox9-high cluster versus the combined population of the remaining Sox9-high clusters to identify cluster-enriched expression programs. Each intersection was performed separately using stage-matched DEG and CUT&RUN peak sets. Spike-in reads were used to calculate per-library scale factors, which were applied to the fragment-level bedGraph files and BigWig coverage tracks. Within each assay, identical software versions, filtering criteria, and analysis parameters were applied across all samples, and GRCm39/GENCODE vM33-based reference resources were used consistently across the two data modalities. The example datasets are available under the accession numbers listed in the key resources table.

## Limitations

This protocol provides a framework for inferring stage- and cluster-resolved Sox9-associated transcriptional regulation in mouse embryonic forelimb buds by integrating scRNA-seq and CUT&RUN data. However, several limitations should be considered when interpreting the results.

First, cluster annotations are based on marker gene expression and do not by themselves establish developmental relationships among the annotated populations. Such relationships require independent experimental validation, such as lineage tracing. Mature chondrocytes are analyzed as a single population in this protocol, although additional transcriptional heterogeneity may be present; in the example dataset, this cluster contained 521 cells, limiting robust subclustering.

Second, the scRNA-seq and CUT&RUN datasets were generated from separate samples, and therefore do not provide a direct single-cell link between Sox9 binding and gene expression. In the example dataset, scRNA-seq was performed using ICR mice, whereas CUT&RUN used B6J Sox9-Multitag knock-in mice; differences in genetic background or sample composition may therefore contribute to differences between the datasets. In addition, scRNA-seq covers E9.5–E13.5, whereas CUT&RUN was performed only at E11.5 and E13.5, limiting the temporal resolution of the integrated analysis.

Third, the integration analysis is restricted to genes associated with promoter-proximal CUT&RUN peaks located within 3 kb of a TSS. This restriction increases confidence in peak-to-gene assignment but excludes distal regulatory elements. Because distal enhancers can mediate a substantial proportion of the tissue-specific activity of Sox9 and other transcription factors, the resulting candidate lists do not represent the complete Sox9 regulatory network. Analysis of distal regulation requires independent evidence linking enhancers to their target promoters, such as chromatin-contact data, perturbation experiments, or validated enhancer annotations.

Overlap between an upregulated DEG and a promoter-associated CUT&RUN gene identifies a candidate Sox9 target gene but does not establish functional causality. A promoter-proximal peak does not by itself demonstrate that Sox9 regulates the associated gene, and the absence of a promoter-proximal peak does not exclude regulation through a distal element. Candidate genes should therefore be interpreted in the context of developmental stage, cell population, peak position, published binding data, and independent functional evidence.

Fourth, the CUT&RUN component depends on a Ty1-tagged Sox9 knock-in mouse and an anti-Ty1 antibody. When adapting this approach to another transcription factor, independently determine whether tag insertion affects protein expression, localization, or function and validate recovery efficiency and signal-to-noise ratio in the target tissue.

Only one Sox9 CUT&RUN library was generated for each developmental stage, preventing assessment of peak reproducibility across independent biological replicates. In addition, only one IgG control library was available, generated from wild-type E11.5 embryos; therefore, no stage-matched IgG control was available for E13.5. The IgG track was used only as an exploratory reference and was not used to define the final peak sets, which were called using a numerical SEACR threshold. Peak specificity was additionally assessed using established Sox9 target loci, published Sox9 ChIP-seq data, and H3K27ac enrichment, but these analyses do not replace validation with independent biological replicates and stage-matched controls. (see Sega et al.^1^).

Finally, the spike-in DNA is added after MNase digestion. Spike-in normalization therefore accounts for post-digestion chromatin recovery, library preparation, and sequencing depth but does not correct for differences in input cell number, antibody binding, MNase digestion, or chromatin recovery efficiency during the CUT&RUN reaction. Spike-in-normalized tracks are useful for displaying coverage on a common scale, but quantitative comparisons of Sox9 binding intensity between developmental stages should therefore be interpreted cautiously.

## Troubleshooting

### Problem 1

Embryos are not synchronized across developmental stages, resulting in large sample-to-sample variability. (Related to before you begin Steps 1–5).

### Potential solution

Keep the timing of mating setup, vaginal plug confirmation, and embryo collection as consistent as possible across experiments. Exclude embryos with clearly delayed development. Prepare multiple litters to account for failed pregnancies or insufficient cell numbers. Variability in developmental stage directly affects cluster composition and candidate Sox9 target identification, so embryo selection should be prioritized.

### Problem 2

Cell clumps remain after forelimb bud dissociation, or a sufficient single-cell suspension cannot be obtained. (Related to Steps 7–25).

### Potential solution

Avoid over-mincing the forelimb buds; cut each limb bud into approximately 10 pieces for E11.5 samples and approximately 20 pieces for E13.5 samples using micro-scissors. Excessive fragmentation causes epithelial-like tissue to form aggregates, reducing dissociation efficiency. During collagenase D treatment, tap the tube gently every 30 min and monitor dissociation. If cell clumps persist, extend the enzymatic reaction by 15–30 min and reassess (do not exceed 2 h total). Passing the suspension through a 70 μm strainer a second time can be effective for clump removal.

### Problem 3

Low viability, excessive debris, or many cell clumps in scRNA-seq samples make them unsuitable for GEM generation. (Related to Steps 75–77).

### Potential solution

Do not fix scRNA-seq samples, and minimize the waiting time between dissociation and library preparation. Keep samples on ice whenever possible and avoid vigorous pipetting. During collagenase D treatment at 37°C with agitation at 1,000 rpm, assess the extent of tissue dissociation every 30 min by gently tapping the samples. Unfixed forelimb tissues generally dissociate more rapidly than fixed tissues; therefore, terminate the collagenase D treatment as soon as sufficient dissociation is achieved and avoid unnecessary prolonged incubation at 37°C.

If debris or clumps are abundant, resuspend the sample gently by pipetting and re-assess; if clumps persist, straining through a smaller pore size than the 70 μm strainer used during dissociation may be considered, bearing in mind the associated cell loss. If viability remains below 70% after optimizing dissociation conditions, either re-prepare the sample or perform dead cell removal on the current suspension and re-assess before proceeding (see Step 76 for the criteria applied at this stage).

### Problem 4

The cell pellet is difficult to see after centrifugation, leading to sample loss.(Related to Steps 32–35).

### Potential solution

Use flat-bottom 1.5 mL low-protein-binding tubes (e.g., Proteosave SS) and confirm that a pellet has formed at the bottom of the tube after each centrifugation. When removing the supernatant, maintain a consistent tube orientation and aspirate from the side opposite to the pellet. If the pellet is not visible, re-centrifuge the sample under the same conditions, or rotate the tube under direct light to locate it.

### Problem 5

Low ConA bead recovery, or bead slurry dries out and destabilizes downstream reactions. (Related to Steps 26–38).

### Potential solution

Wait until beads have fully cleared on the magnet before removing the supernatant. After washing, minimize the interval before the next buffer addition to prevent bead drying. Prepare the required volumes of activation buffer and wash buffer in advance and perform the operations continuously. Maintaining consistent bead volumes across samples is also important.

### Problem 6

Weak CUT&RUN signal or high background in the IgG negative control. (Related to Steps 38–59).

### Potential solution

Strictly standardize antibody amount, pAG-MNase amount, and the reaction time and temperature after CaCl_2_ addition across all samples. Prepare digitonin-containing buffers and protease inhibitor-supplemented buffers immediately before use. When an IgG negative control is included, process it in parallel under the same conditions as the target sample. If problems occur, prioritize re-evaluation of antibody reaction uniformity, pAG-MNase reaction time, and washing sufficiency. If the signal is weaker than expected in Ty1-tagged Sox9 samples, reconfirm embryo genotyping and the consistency of developmental stage at sample collection.

### Problem 7

Low yield of CUT&RUN DNA or library, or abnormal size distribution on TapeStation. (Related to Steps 60–69).

### Potential solution

Avoid over-drying SPRIselect beads after washing. Proceed to the elution step promptly after removing 80% ethanol. If adapter dimers or excessive high-molecular-weight fragments are prominent on TapeStation, re-evaluate input DNA amount, PCR amplification conditions, and SPRI cleanup parameters. If strong high-molecular-weight signal is also observed in the IgG negative control, background elevation may originate from upstream CUT&RUN conditions.

### Problem 8

Very limited or difficult-to-interpret overlap in scRNA-seq and CUT&RUN integration analysis. (Related to Steps 91–103).

### Potential solution

First, check for gene symbol inconsistencies and peak-to-gene assignment rules. This protocol uses GRCm39 for both scRNA-seq and CUT&RUN, with GENCODE vM33 gene models for peak annotation, so coordinate systems are consistent; however, the Cell Ranger reference applies filtering to the GENCODE gene set, so a small number of genes annotated in the full release may be absent from the scRNA-seq gene–cell matrix. Verify which genes are present in one dataset but not the other rather than assuming complete correspondence. Confirm that the promoter window used for peak-to-gene assignment (TSS ± 3 kb in this protocol) is appropriate for the factor of interest; a narrow window will exclude distal regulatory elements and reduce the apparent overlap. Interpret candidate Sox9 target genes with the understanding that they are inferred from the overlap of peak-associated genes and cluster-enriched genes, not from direct single-cell matching. If needed, visualize representative loci on a genome browser to aid interpretation.

### Problem 9

Control-based peak calling returns an implausibly large number of peaks. (Related to Step 99).

### Potential solution

Confirm that the correct control bedGraph and SEACR argument order are being used, and inspect the target and control coverage tracks at established target loci. If control-based peak calling produces an implausibly broad peak set, compare the result with numerical-threshold peak calling. In the example dataset, the final peak sets were generated consistently using a numerical threshold of 0.001 rather than the IgG control.

## Resource availability

### Lead contact

Further information and requests for resources and reagents should be directed to and will be fulfilled by the lead contact, Hiroshi Asahara (asahara.syst@tmd.ac.jp).

### Technical contact

Technical questions on executing this protocol should be directed to and will be answered by the technical contact, Kohei Kita (kcjk1188@tmd.ac.jp).

### Materials availability

The Sox9-Multitag mouse line used in this protocol is available from the lead contact upon reasonable request.

### Data and code availability

• The analyzed data generated in this study have been deposited in the DDBJ database. Accession numbers are listed in the key resources table.
• All code required to reproduce the analyses described in this protocol is provided in the step-by-step method details. No additional original code was used.
• Any additional information required to reanalyze the data reported in this paper is available from the lead contact upon request.

## Acknowledgments

This research was supported by AMED under grant nos. JP22gm0010009, JP22ama121045, JP24jf0126010, JP24gm2010002, JP25ek0109836, and JP256f0137003 to H.A. This research was also supported by the JST Program for Co-Creating Startup Ecosystem (grant no. JPMJSF2313), JSPS KAKENHI (grant nos. JP26K21770 to H.A. and JP25K23806 to Y.U.), and the National Institutes of Health (NIH) (grant no. R01AR080127 to H.A.).

We thank all the staff of the Department of Systems Biomedicine at the Institute of Science Tokyo for their support and advice. The graphical abstract was partially created in BioRender. K.K. (2026) https://BioRender.com/ah6s5fk.

## Author contributions

Conceptualization, K.K., M.S., Y.U., and H.A.; methodology, K.K., M.S., T.C., T.M., and H.A.; validation, K.K., M.S., and T.C.; formal analysis, M.S. and Y.U.; investigation, K.K. and M.S.; resources, T.M.; data curation, Y.U.; writing – original draft preparation, K.K.; writing – review and editing, K.K., M.S., Y.U., T.C., T.M., and H.A.; visualization, K.K., M.S., and Y.U.; supervision, H.A.; project administration, H.A.; funding acquisition, H.A.

## Declaration of interests

The authors declare no competing interests.

## Declaration of generative AI and AI-assisted technologies in the manuscript preparation process

During the preparation of this work, the authors used Claude (Anthropic) to improve the clarity and language of the manuscript. The authors reviewed and edited the output as needed and take full responsibility for the content of the published article.

## References

[1] Sega M., Uchida Y., Chiba T., Matsushima T., Kita K., Miyasaka N., Asahara H. (2026). Stage- and cluster-specific regulation of chondrogenic gene programs by Sox9 in mouse embryonic limb buds. iScience.

[2] Skene P.J., Henikoff S. (2017). An efficient targeted nuclease strategy for high-resolution mapping of DNA binding sites. eLife.

[3] Hao Y., Stuart T., Kowalski M.H., Choudhary S., Hoffman P., Hartman A., Srivastava A., Molla G., Madad S., Fernandez-Granda C., Satija R. (2024). Dictionary learning for integrative, multimodal and scalable single-cell analysis. Nat. Biotechnol..

[4] Andrews S. (2010). FastQC: a quality control tool for high throughput sequence data. Babraham Bioinformatics. https://www.bioinformatics.babraham.ac.uk/projects/fastqc/.

[5] Langmead B., Salzberg S.L. (2012). Fast gapped-read alignment with Bowtie 2. Nat. Methods.

[6] Danecek P., Bonfield J.K., Liddle J., Marshall J., Ohan V., Pollard M.O., Whitwham A., Keane T., McCarthy S.A., Davies R.M., Li H. (2021). Twelve years of SAMtools and BCFtools. GigaScience.

[7] Ramírez F., Dündar F., Diehl S., Grüning B.A., Manke T. (2014). deepTools: a flexible platform for exploring deep-sequencing data. Nucleic Acids Res..

[8] Quinlan A.R., Hall I.M. (2010). BEDTools: a flexible suite of utilities for comparing genomic features. Bioinformatics.

[9] Meers M.P., Tenenbaum D., Henikoff S. (2019). Peak calling by Sparse Enrichment Analysis for CUT&RUN chromatin profiling. Epigenetics Chromatin.

[10] Heinz S., Benner C., Spann N., Bertolino E., Lin Y.C., Laslo P., Cheng J.X., Murre C., Singh H., Glass C.K. (2010). Simple combinations of lineage-determining transcription factors prime cis-regulatory elements required for macrophage and B cell identities. Mol. Cell.

[11] Yu G., Wang L.G., He Q.Y. (2015). ChIPseeker: an R/Bioconductor package for ChIP peak annotation, comparison and visualization. Bioinformatics.

[12] Yea J.H., Onggo S.E.S., Dewagan S., Chen K., Young K.K., Cahan P. (2026). Protocol to Investigate the Effects of Localized Gene Expression Disruption on Mouse Limb Development via Direct siRNA Injection and Explant Culture. STAR Protoc..

[13] Janssens D.H., Wu S.J., Sarthy J.F., Meers M.P., Myers C.H., Olson J.M., Ahmad K., Henikoff S. (2018). Automated in situ chromatin profiling efficiently resolves cell types and gene regulatory programs. Epigenetics Chromatin.

